# Capture of Totipotency in Mouse Embryonic Stem Cells in the Absence of *Pdzk1*

**DOI:** 10.1002/advs.202408852

**Published:** 2024-12-04

**Authors:** Wenhao Zhang, Yiding Zhao, Zhe Yang, Jing Yan, Haisong Wang, Shaochen Nie, Qingshen Jia, Dan Ding, Chao Tong, Xiao‐Ou Zhang, Qian Gao, Ling Shuai

**Affiliations:** ^1^ State Key Laboratory of Medicinal Chemical Biology and College of Pharmacy Nankai Animal Resources Center and Reproductive Regulation Institute of Transplantation Medicine Nankai University Tianjin 300350 China; ^2^ Shanghai Key Laboratory of Maternal and Fetal Medicine Clinical and Translational Research Center of Shanghai First Maternity and Infant Hospital Frontier Science Center for Stem Cell Research School of Life and Science and Technology Tongji University Shanghai 200092 China; ^3^ Reproductive Medical Center Henan Key Laboratory of Reproduction and Genetics The First Affiliated Hospital of Zhengzhou University Henan 450052 China; ^4^ National Clinical Research Center for Child Health and Disorders Ministry of Education Key Laboratory of Child Development and Disorders Children’s Hospital of Chongqing Medical University Chongqing 401122 China; ^5^ Tianjin Key Laboratory of Animal and Plant Resistance College of Life Sciences Tianjin Normal University Tianjin 300387 China

**Keywords:** blastoids, ESCs, *Pdzk1*, totipotent‐like state

## Abstract

Totipotent cells can differentiate into three lineages: the epiblast, primitive endoderm, and trophectoderm. Naturally, only early fertilized embryos possess totipotency, and they lose this ability as they develop. The expansion of stem cell differentiation potential has been a hot topic in developmental biology for years, particularly with respect to the generation totipotent‐like stem cells. Here, the study describes the establishment of totipotency in embryonic stem cells (ESCs) via the deletion of a single gene, *Pdzk1*. *Pdzk1*‐knockout (KO) ESCs substantially contribute to the fetus, placenta, and yolk sac in chimera assays but can also self‐organize to form standard blastocyst‐like structures containing the three lineages efficiently; thus, they exhibit full developmental potential as early blastomeres. Single‐cell transcriptome and bulk RNA‐seq comprehensive analyses revealed that *Pdzk1*‐KO activates several lineage inducers (*C1qa*, *C1qb*, *Fgf5*, and *Cdx2*) to break down barriers between embryonic and extraembryonic tissues, making these lineages switch smoothly and resulting in a totipotent‐like state. This versatile and scalable system provides a robust experimental model for differentiation potency and cell fate studies.

## Graphical abstract

This work provides a new strategy to capture totipotency in ESCs by deletion of *Pdzk1*. *Pdzk1*‐null ESCs can integrate to fetus, placenta, and yolk sac tissues easily in chimera experiment, and also can self‐organize to form blastocyst‐like structures efficiently. The developmental potentials of *Pdzk1*‐KO ESCs are mainly through up‐regulating some lineage inducers, giving a new view to know totipotency.

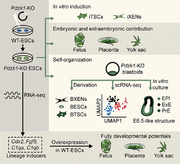

## 1 Introduction

Totipotency is the ability of a single cell to give rise to an entire embryo and its extraembryonic counterparts and is restricted to one‐ and two‐cell‐stage fertilized embryos in mice.^[^ ^1^ ^]^ Thereafter, the differentiation potential of developing blastomeres becomes gradually limited during embryogenesis. Early embryos develop into three lineages, namely, the epiblast (EPI), primitive endoderm (PrE), and trophectoderm (TE), which are precursors of all embryonic germ layers, the extraembryonic yolk sac and the placenta.^[^ ^2^ ^]^ However, there are strict boundaries among the three lineages in mice, which makes the natural transition from one lineage to others quite difficult. The key barriers among them in mice remain largely unknown; thus, how to regulate cell fate transition among them is a hot topic in developmental biology. Although EPI, PrE and TE exist transiently in the developing embryo, stem cells can be derived from them separately via defined culture systems (embryonic stem cells (ESCs), extraembryonic endoderm (XEN) cells and trophoblast stem cells (TSCs)), with self‐renewal and differentiation potential.^[^ ^3^, ^4^, ^5^ ^]^ These stem cells are ideal tools for investigating these strict lineage restrictions in vitro. ESCs can spontaneously give rise to XENs in culture via defined factors.^[^ ^6^, ^7^, ^8^ ^]^ Gene editing of specific transcription factors or epigenetic regulators could promote the murine ESC‐to‐TSC transition.^[^ ^9^, ^10^, ^11^, ^12^, ^13^, ^14^, ^15^, ^16^, ^17^, ^18^ ^]^

Although the differentiation potential of these three types of stem cells is restricted to their own lineages, a small and transient 2‐cell embryo (2C)‐like subpopulation exists in mouse ESCs, with both embryonic and extraembryonic developmental potential.^[^ ^19^ ^]^ Recently, several totipotent‐like stem cells have been established, including totipotent blastomere‐like cells (TBLCs),^[^ ^20^ ^]^ totipotent‐like stem cells (TLSCs),^[^ ^21^ ^]^ chemically induced totipotent stem cells (ciTotiSCs)^[^ ^22^ ^]^ and totipotent potential stem cells (TPSCs),^[^ ^23^ ^]^ which have the ability to differentiate into extraembryonic and embryonic lineages. However, the barriers that prevent the transition among the three lineages remain unknown. In a previous study, we performed genetic screening to reveal key blockers for the ESC‐to‐TSC transition via a haploid system. The top 10 genes were ranked according to the score and insertion frequency of *piggyBac* (PB), the top 2 candidates (*Catip* and *Dyrk1a*) of which function in restricting the plasticity of ESCs.^[^ ^17^ ^]^ *Pdzk1* was the 3rd candidate gene in the list and encodes a scaffold protein that connects plasma membrane proteins and regulatory components, regulating their surface expression in the apical domains of epithelial cells.^[^ ^24^ ^]^ In addition, PDZ domains are involved in many biological functions, including protein scaffolding, the organization of ion channels, and signal transduction. PDZK1 contains four PDZ domains and has been identified in the kidney, pancreas, liver, gastrointestinal tract, and adrenal cortex.^[^ ^25^ ^]^ To date, the role of Pdzk1 in regulating the plasticity of ESCs has not been studied.

Here, we assessed whether the deletion of *Pdzk1* facilitates the development of a more plastic state in ESCs. Their developmental potential is assessed by many assays, including chimeric experiments and the formation of blastocyst‐like structures. In addition, we comprehensively compared our stem cells with WT‐ESCs at the single‐cell level and bulk RNA level to identify critical inducers that breakdown lineage barriers between embryonic and extraembryonic tissues.

## 2 Results

### 2.1 *Pdzk1*‐Null Promotes ESC Tripotency to ESCs, TSCs, and XENs

**Figure 1:**
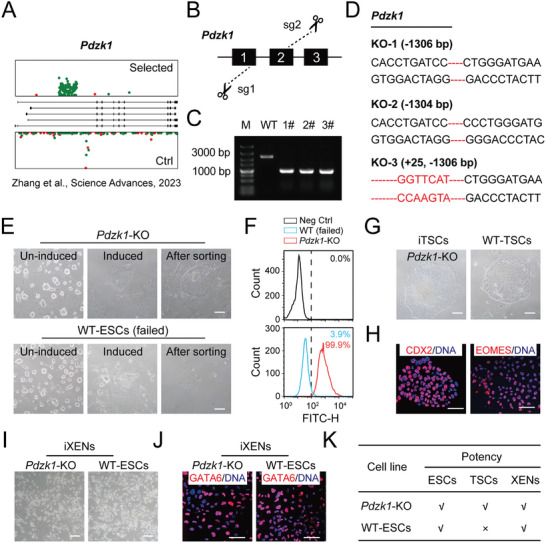
Extended potency of *Pdzk1*‐KO ESCs. A) Sense (red) and antisense (green) insertions of PB in the *Pdzk1* gene body from a previous screen of iTSCs via a haploid system. The rectangles indicate the exons. B) Schematic diagram of the strategy for deleting *Pdzk1* in WT‐ESCs via the CRISPR/Cas9 system. C) Genotype PCR results of *Pdzk1*‐KO subclones. D) Genotyping of *Pdzk1*‐KO ESCs via DNA sequencing. E) Morphological images of the induced cell cultures of *Pdzk1*‐KO ESCs and WT‐ESCs before and after cell sorting with a TSC‐specific antibody (CDCP1). Scale bar, 100 µm. F) Percentages of CDCP1+ cells in induced cultures of *Pdzk1*‐KO ESCs and WT‐ESCs. G) Morphological images of *Pdzk1*‐KO iTSCs and WT‐TSCs. Scale bar, 100 µm. H) Immunofluorescence of TSC‐specific markers (CDX2 and EOMES) in *Pdzk1*‐KO iTSCs and WT‐TSCs. DNA was stained with Hoechst 33342. Scale bar, 50 µm. I) Morphological images of *Pdzk1*‐KO iXENs and WT‐ESC‐derived iXENs. Scale bar, 100 µm. J) Immunofluorescence of the XEN‐specific marker (GATA6) in *Pdzk1*‐KO iXENs and WT‐ESC iXENs. DNA was stained with Hoechst 33342. Scale bar, 50 µm. K) Summary of the potencies of *Pdzk1*‐KO ESCs and WT‐ESCs.

Conversion of mouse ESCs to TSCs or XENs is difficult due to lineage restrictions. According to our previous ESC‐to‐TSC haploid screening,^[^ ^17^ ^]^ many PB insertions located in the *Pdzk1* gene body are enriched (**Figure** **1**A). We investigated whether the deletion of *Pdzk1* could increase the potential of ESCs to differentiate into TSCs or XENs. Thus, we designed specific Cas9‐green fluorescent protein (GFP) knockout (KO) vectors and transfected them into mouse wild‐type (WT)‐ESCs via electroporation (Figure 1B). Among the transfected subclones, three KO (‐/‐) lines were identified by genotyping via PCR and sequencing (Figure 1C,D). We randomly chose one *Pdzk1*‐KO ESC line (KO‐1, 1306 bp/1306 bp deletion) for further experiments. To examine whether *Pdzk1*‐KO affects genome integrity and pluripotency, we assessed the karyotypes of the *Pdzk1*‐KO ESCs and WT‐ESCs and found that there were not chromosomal abnormalities in both of them (Figure S1A,B, Supporting Information). The immunofluorescence results revealed that *Pdzk1*‐KO ESCs highly expressed pluripotent markers (OCT4 and NANOG) and were unaffected by gene editing (Figure S1C, Supporting Information). To assess the differentiation potential of the three germ layers, we injected ≈1 × 10^7^ *Pdzk1*‐KO ESCs into the limbs of severe combined immunodeficiency (SCID) male mice. The *Pdzk1*‐KO ESCs could form standard teratomas containing tissues representative of the three germ layers, similar to WT‐ESCs (Figure S1D, Supporting Information). Thus, the deletion of *Pdzk1* in ESCs did not jeopardize pluripotency.

To address whether *Pdzk1*‐KO ESCs can transition to extraembryonic lineages in a defined culture system, we cultured them in TSC medium to induce the conversion of TSCs. Typically, induced TSCs (iTSCs) could be generated in the *Pdzk1*‐KO group, which were sorted and enriched with a TSC‐specific flow cytometric antibody (CDCP1), whereas the WT‐ESC group could not undergo this process (Figure 1E,F). The *Pdzk1*‐KO iTSCs resembled WT‐TSCs in morphology (Figure 1G) and expressed TSC‐specific markers (CDX2 and EOMES), as indicated by immunostaining (Figure 1H). Given that mouse ESCs can generate extraembryonic endoderm (XEN) cell lines in a defined culture system,^[^ ^6^ ^]^ we evaluated our *Pdzk1*‐KO ESCs and WT‐ESCs under the same conditions to induce XENs. Both *Pdzk1*‐KO ESCs and WT‐ESCs were able to form standard XENs after induction (Figure 1I) and expressed an XEN‐specific marker (GATA6) after long‐term passage (Figure 1J). Taken together, these findings indicate that *Pdzk1*‐KO ESCs are tripotent to ESCs, TSCs, and XENs, whereas WT‐ESCs are only bipotent to ESCs and XENs. Compared with WT‐ESCs, *Pdzk1*‐KO ESCs exhibited increased developmental potential in vitro.

### 2.2 *Pdzk1*‐KO ESCs have Developmental Potential for Embryonic and Extraembryonic Tissues In Vivo

**Figure 2:**
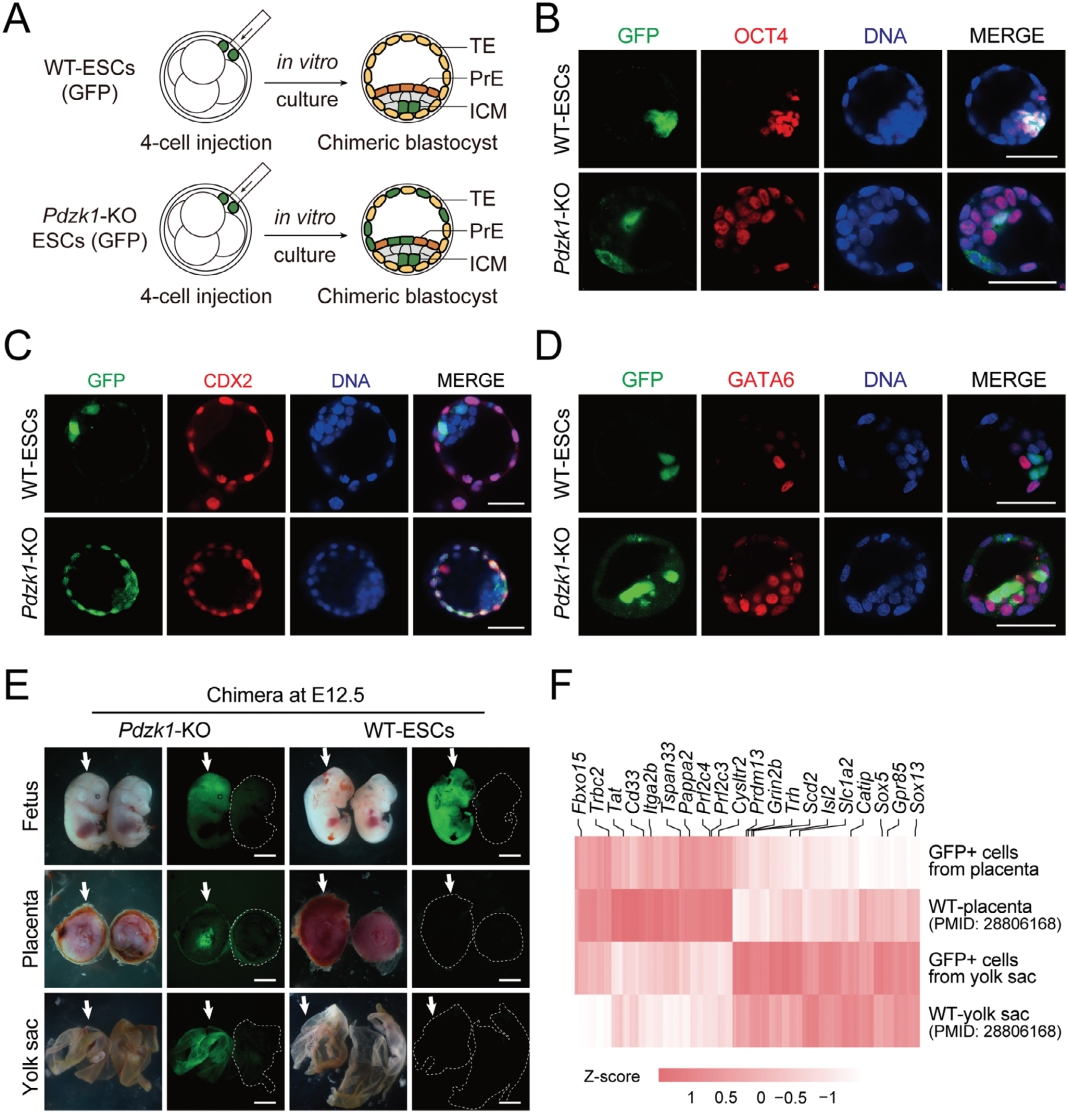
Advanced developmental potential of *Pdzk1*‐KO ESCs in chimeras. A) Schematic overview of the developmental contribution of microinjection of *Pdzk1*‐KO ESCs and WT‐ESCs into chimeric embryos. B) Immunofluorescence of OCT4 (red) in chimeric blastocysts derived from GFP‐labeled (green) *Pdzk1*‐KO ESCs and WT‐ESCs. DNA was stained with Hoechst 33342 (blue). Scale bar, 50 µm. C) Immunofluorescence of CDX2 (red) in chimeric blastocysts derived from GFP‐labeled (green) *Pdzk1*‐KO ESCs and WT‐ESCs. DNA was stained with Hoechst 33342 (blue). Scale bar, 50 µm. D) Immunofluorescence of GATA6 (red) in chimeric blastocysts derived from GFP‐labeled (green) *Pdzk1*‐KO ESCs and WT‐ESCs. DNA was stained with Hoechst 33342 (blue). Scale bar, 50 µm. E) Images of E12.5 chimeras (including fetus, placenta and yolk sac) derived from GFP‐labeled *Pdzk1*‐KO ESCs, with GFP‐labeled WT‐ESC‐derived chimeras used as a control. The white arrowheads indicate the fetus, placenta and yolk sac from the chimeric embryo. Scale bar, 5 mm. F) Heatmap representation of genes predominantly expressed in GFP+ cells from the chimeric placenta and chimeric yolk sac. The WT‐placenta and WT‐yolk sac data were downloaded from a previous study (Andergassen et al., 2017, Elife). Representative lineage markers are indicated.

To further confirm the advanced developmental potential of *Pdzk1*‐KO ESCs, we performed a chimeric assay by microinjecting GFP‐labeled donor ESCs into 4‐cell‐stage embryos (Figure S2A, Supporting Information; Figure 2A). The immunostaining results of chimeric blastocysts indicated that *Pdzk1*‐KO ESCs could contribute to EPI (OCT4+), PrE (GATA6+), and TE (CDX2+) tissues easily, whereas WT‐ESCs were only able to integrate into the EPI region (**Figure** **2**B–D). *Pdzk1*‐KO ESCs exhibited a totipotent‐like state with bidirectional potential to both embryonic and extraembryonic counterparts. A stricter assessment of the totipotent state is the subsequent development of chimeras in vivo (Figure S2B, Supporting Information).^[^ ^20^ ^]^ Therefore, we transferred GFP‐labeled *Pdzk1*‐KO ESC and WT‐ESC chimeric embryos to the oviducts of pseudopregnant mice separately. At embryonic day (E) 12.5, *Pdzk1*‐KO ESCs contributed very clearly to the fetus, placenta and yolk sac, whereas WT‐ESCs only integrated into the fetus (Figure 2E). By precisely quantifying the cooperation efficiency of donor cells via FACS, we found that GFP‐positive (GFP+) cells derived from *Pdzk1*‐KO ESCs contributed to 42.3% of the fetus, 5.1% of the placenta, and 51.4% of the yolk sac (Figure S2C, Supporting Information). In parallel, immunofluorescence analysis of the *Pdzk1*‐KO ESC‐derived E12.5 chimeric placenta further confirmed that *Pdzk1*‐KO ESCs had developmental potential in the functional placenta (GCM1‐, PROLIFERIN‐, TPBPA‐, and TFAP2C‐positive) (Figure S2D, Supporting Information). Next, we sorted the GFP+ cells from the chimeric placenta and the chimeric yolk sac for RNA‐seq and further analysis and compared them with those from the WT‐placenta and the WT‐yolk sac.^[^ ^26^ ^]^ The GFP+ cells from the chimeric placenta highly expressed some placental marker genes (such as *Prl2c3* and *Prl2c4*), similar to those in the WT‐placenta, whereas the GFP+ cells from the chimeric yolk sac highly expressed some yolk sac marker genes (such as *Prdm13*, *Sox5*, and *Sox13*), similar to those in the WT‐yolk sac (Figure 2F).

### 2.3 Plasticity of *Pdzk1*‐KO ESCs in Generating Blastoids

**Figure 3:**
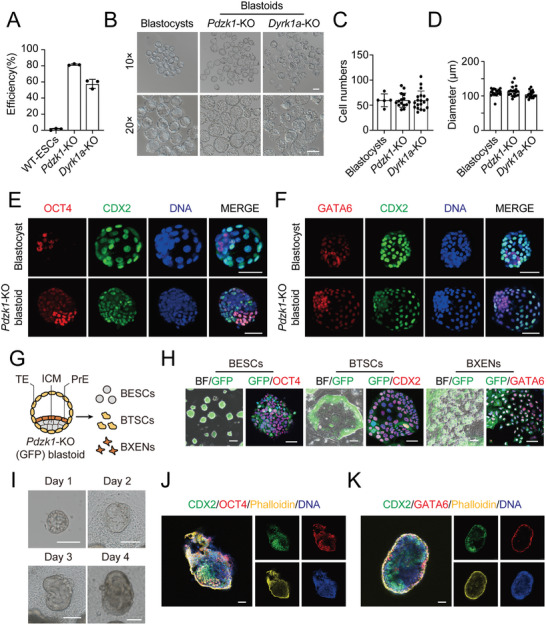
Generation and development of *Pdzk1*‐KO ESC‐derived blastoids. A) Blastoid formation efficiencies of *Pdzk1*‐KO ESCs and *Dyrk1a*‐KO ESCs, with WT‐ESCs serving as a negative control. B) Images of *Pdzk1*‐KO blastoids, *Dyrk1a*‐KO blastoids and E4.5 mouse blastocysts. Scale bar, 100 µm. C) Total number of cells in each E4.5 mouse blastocyst (n = 5) and each blastoid (*Pdzk1*‐KO, n = 15; *Dyrk1a*‐KO, n = 19). D) The diameter of each E4.5 mouse blastocyst (n = 19) and each blastoid (*Pdzk1*‐KO, n = 19; *Dyrk1a*‐KO, n = 19) separately. E) Immunofluorescence of OCT4 (red) and CDX2 (green) in *Pdzk1*‐KO blastoids. WT blastocysts were used as controls. DNA was stained with Hoechst 33342 (blue). Scale bar, 50 µm. F) Immunofluorescence of GATA6 (red) and CDX2 (green) in *Pdzk1*‐KO blastoids. WT blastocysts were used as controls. DNA was stained with Hoechst 33342 (blue). Scale bar, 50 µm. G) Schematic overview of the establishment of BESCs, BTSCs, and BXENs derived from GFP‐labeled blastoids. H) GFP and immunofluorescence images of BESCs, BTSCs, and BXENs derived from *Pdzk1*‐KO blastoids with specific markers. Scale bar, GFP images, 100 µm; Immunofluorescence images, 50 µm. I) Images of in vitro cultured (IVC) blastoids on Days 1–4. Scale bars, 100 µm. J) Immunofluorescence of OCT4 and CDX2 in IVC blastoids on Day 4. Scale bar, 50 µm. K) Immunofluorescence of CDX2 and GATA6 in IVC blastoids on Day 4. Scale bar, 50 µm.

Whether *Pdzk1*‐KO ESCs can generate structures that mimic mouse blastocyst development in vitro is unknown and fascinating. To this end, we developed a 3D culture system to form blastocyst‐like structures with *Pdzk1*‐KO ESCs according to our previous protocol (Figure S3A, Supporting Information), with previously reported totipotent‐like stem cells (*Dyrk1a*‐KO ESCs) used as a positive control.^[^ ^17^ ^]^ After ≈10 days, both *Pdzk1*‐KO ESCs and *Dyrk1a*‐KO ESCs generated blastoids with a standard morphology of blastocyst structures, whereas WT‐ESCs did not have this potential (Figure S3B, Supporting Information). The *Pdzk1*‐KO ESCs had nearly 80% blastoid formation efficiency, which was greater than that of *Dyrk1a*‐KO ESCs (≈60%, as determined by quantification) (**Figure** **3**A). The *Pdzk1*‐KO blastoids and *Dyrk1a*‐KO blastoids contained inner cell mass (ICM)‐like and trophectoderm‐like compartments with a visible cavity and had similar morphologies to those of the WT‐blastocysts (Figure 3B). Thereafter, we evaluated the quality of these blastoids. The cell numbers and dimensions of *Pdzk1*‐KO blastoids were close to those of WT blastocysts at E4.5 and *Dyrk1a*‐KO blastoids (Figure 3C,D). The immunofluorescence results revealed OCT4+, GATA6+ and CDX2+ cells in *Pdzk1*‐KO blastoids, demonstrating that they contained all lineages (EPI, PrE, and TE) of a blastocyst (Figure 3E,F). To further investigate their properties, we plated GFP‐labeled *Pdzk1*‐KO blastoids (Figure S3C, Supporting Information) into different culture medium to derive blastoid‐ESCs (BESCs), blastoid‐TSCs (BTSCs) and blastoid‐XENs (BXENs) separately (Figure 3G). The immunofluorescence results revealed that BESCs, BTSCs, and BXENs were double positive for GFP and specific markers (OCT4, CDX2, and GATA6, respectively) (Figure 3H).

To ascertain whether *Pdzk1*‐KO blastoids could further develop into structures similar to those of peri‐implantation embryos,^[^ ^27^, ^28^ ^]^ we cultured the blastoids for another 4 days in an in vitro culture (IVC) system. On IVC Day 4, the outgrowths of *Pdzk1*‐KO blastoids seemed to resemble those of E6.5 embryos morphologically (Figure 3I), containing segregated OCT4+, GATA6+, and CDX2+ cells (Figure 3J,K). These findings demonstrated that *Pdzk1*‐KO blastoids could self‐organize into structures with features of peri‐implantation mouse embryos. A more stringent test for the development of blastoids was to detect whether they could develop to the postimplantation stage in utero. Therefore, we transferred GFP‐labeled *Pdzk1*‐KO blastoids into pseudopregnant mice at 2.5 days postcoitum (dpc) and evaluated them at 6.5 dpc (Figure S3D, Supporting Information). Consistent with previous studies,^[^ ^17^, ^18^, ^21^, ^23^, ^29^, ^30^, ^31^, ^32^ ^]^ *Pdzk1*‐KO blastoids were able to implant and generate deciduae instead of standard E6.5 embryos in vivo (Figure S3E, Supporting Information). We also attempted to treat blastoids with HB‐EGF, which can promote blastocyst growth, zona hatching and trophoblast development.^[^ ^33^ ^]^ Unfortunately, HB‐EGF could still not rescue the development failure of blastoids in vivo (Figure S3E,F, Supporting Information). Nevertheless, all the results suggested that *Pdzk1*‐KO blastoids can efficiently form typical blastocyst‐like structures with implantation ability and further developmental potential.

### 2.4 *Pdzk1*‐KO Blastoid Transcriptomes Globally Resemble those of Mouse Preimplantation Embryos

**Figure 4:**
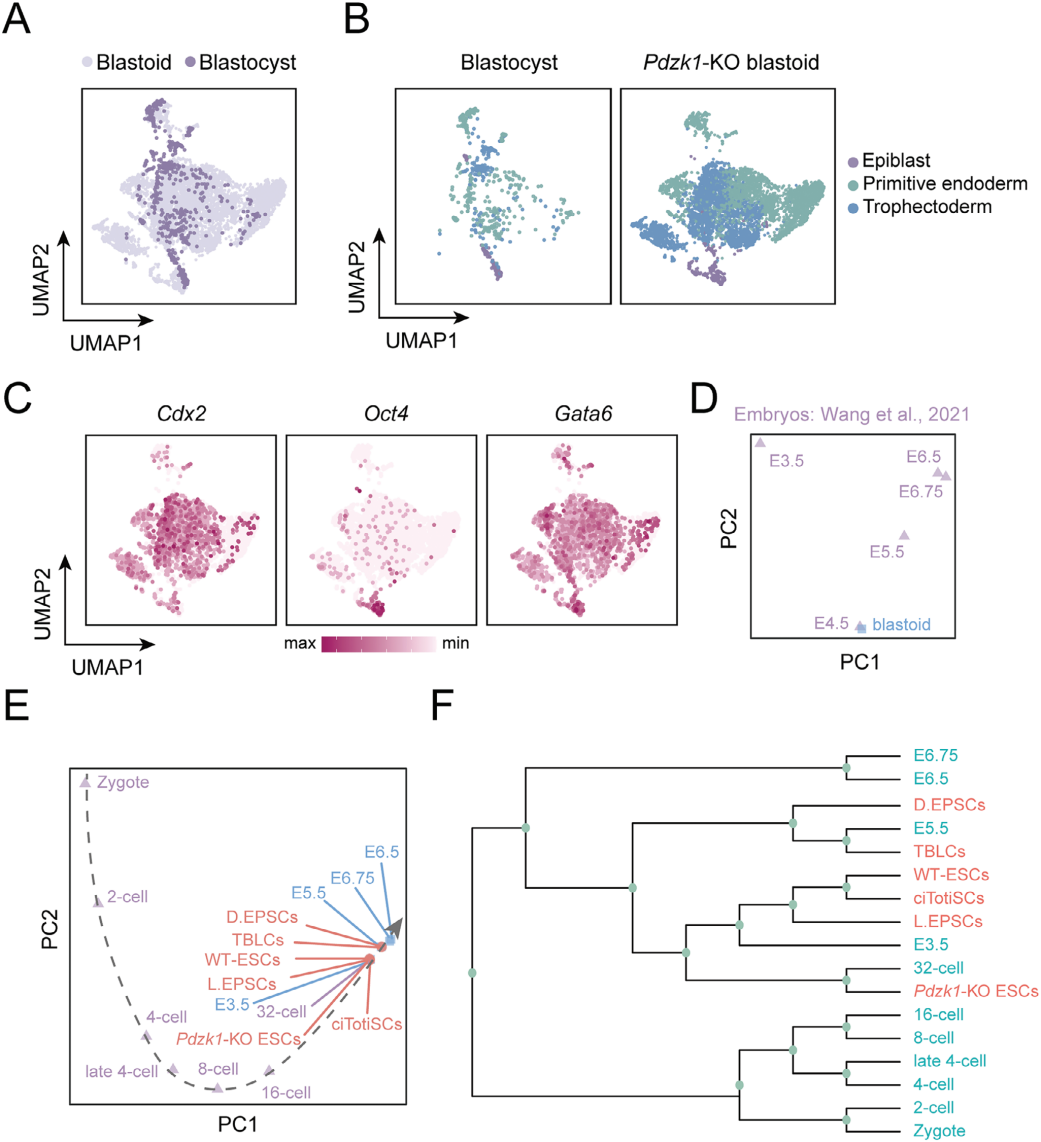
Single‐cell transcriptome analysis of *Pdzk1*‐KO blastoids and *Pdzk1*‐KO ESCs. A) UMAP plots of *Pdzk1*‐KO blastoids and WT blastocysts. B) Comparison of cells derived from *Pdzk1*‐KO blastoids and WT blastocysts. This panel shows lineage assignments: epiblast (purple), primitive endoderm (green) and trophectoderm (blue). C) The expression of representative marker genes for epiblast (*Oct4*), primitive endoderm (*Gata6*) and trophectoderm (*Cdx2*) in *Pdzk1*‐KO blastoids at the single‐cell level. D) Principal component analysis (PCA) of *Pdzk1*‐KO blastoids compared with published datasets of mouse embryos. The embryonic stages are indicated. E) PCA of *Pdzk1*‐KO ESCs, WT‐ESCs, ciTotiSCs, TBLCs, L. EPSCs, D. EPSCs, TBLCs and mouse embryos at different stages at the single‐cell level. F) Hierarchical clustering analysis of *Pdzk1*‐KO ESCs, WT‐ESCs, ciTotiSCs, TBLCs, L. EPSCs, D. EPSCs, TBLCs and mouse embryos at different stages at the single‐cell level.

To determine the transcriptional identity of *Pdzk1*‐KO blastoids, we performed single‐cell RNA sequencing (scRNA‐seq) via the 10x genomics technique. Approximately 6028 *Pdzk1*‐KO blastoid cells were compared with published single‐cell transcriptomes derived from mouse blastocysts (E3.5 and E4.5).^[^ ^29^, ^34^, ^35^ ^]^ Comparative analysis via the Seurat^[^ ^36^ ^]^ package revealed that the cells from *Pdzk1*‐KO blastoids largely overlapped with the published blastocysts, suggesting that the transcriptome of *Pdzk1*‐KO blastoids was similar to that of blastocysts (**Figure** **4**A). Importantly, the cell populations of EPI, PrE, and TE in *Pdzk1*‐KO blastoids transcriptionally resembled those in blastocysts (Figure 4B). Among the 6028 analyzed cells from the blastoids, 302 cells were *Oct4*+ (EPI), 3128 cells were *Gata6*+ (PrE), and 2598 cells were *Cdx2*+ (TE) (Figure 4C). To assess the temporal properties of *Pdzk1*‐KO blastoids, we compared our data with published datasets of mouse embryos at the indicated stages.^[^ ^37^ ^]^ By projecting blastoids onto an axis of developmental time generated from embryo‐derived cells, we found that *Pdzk1*‐KO blastoids most resembled cells from E4.5 embryos (Figure 4D).

To address the properties of *Pdzk1*‐KO ESCs, we also performed scRNA‐seq. Approximately 4295 *Pdzk1*‐KO ESCs and WT‐ESCs were extracted from our datasets. scRNA‐seq‐based principal component analysis (PCA) combined with single‐cell transcriptomes of published mouse embryos and totipotent‐like cells^[^ ^20^, ^22^, ^34^, ^35^, ^37^ ^]^ revealed that *Pdzk1*‐KO ESCs showed somewhat greater similarity to 32‐cell mouse embryos than to E3.5 embryos, whereas other cells were close to E3.5‐E5.5 embryos (Figure 4E,F; Figure S4A, Supporting Information). Some representative 32‐cell marker genes (*Klf5* (86.9%), *Slc7a1* (67.1%), *Kdelr3* (25.0%), *Serpine2* (16.8%), *Egr1* (60.9%), *Manf* (97.3%), *Htra1* (24.4%), and Lama1 (37.9%) were dominant in most populations of *Pdzk1*‐KO ESCs (Figure S4C, Supporting Information), which might explain their plasticity in generating blastoids resembling blastocysts.

### 2.5 *Pdzk1*‐KO Activates Totipotent‐Like States through the Upregulation of Key Lineage Inducers

**Figure 5:**
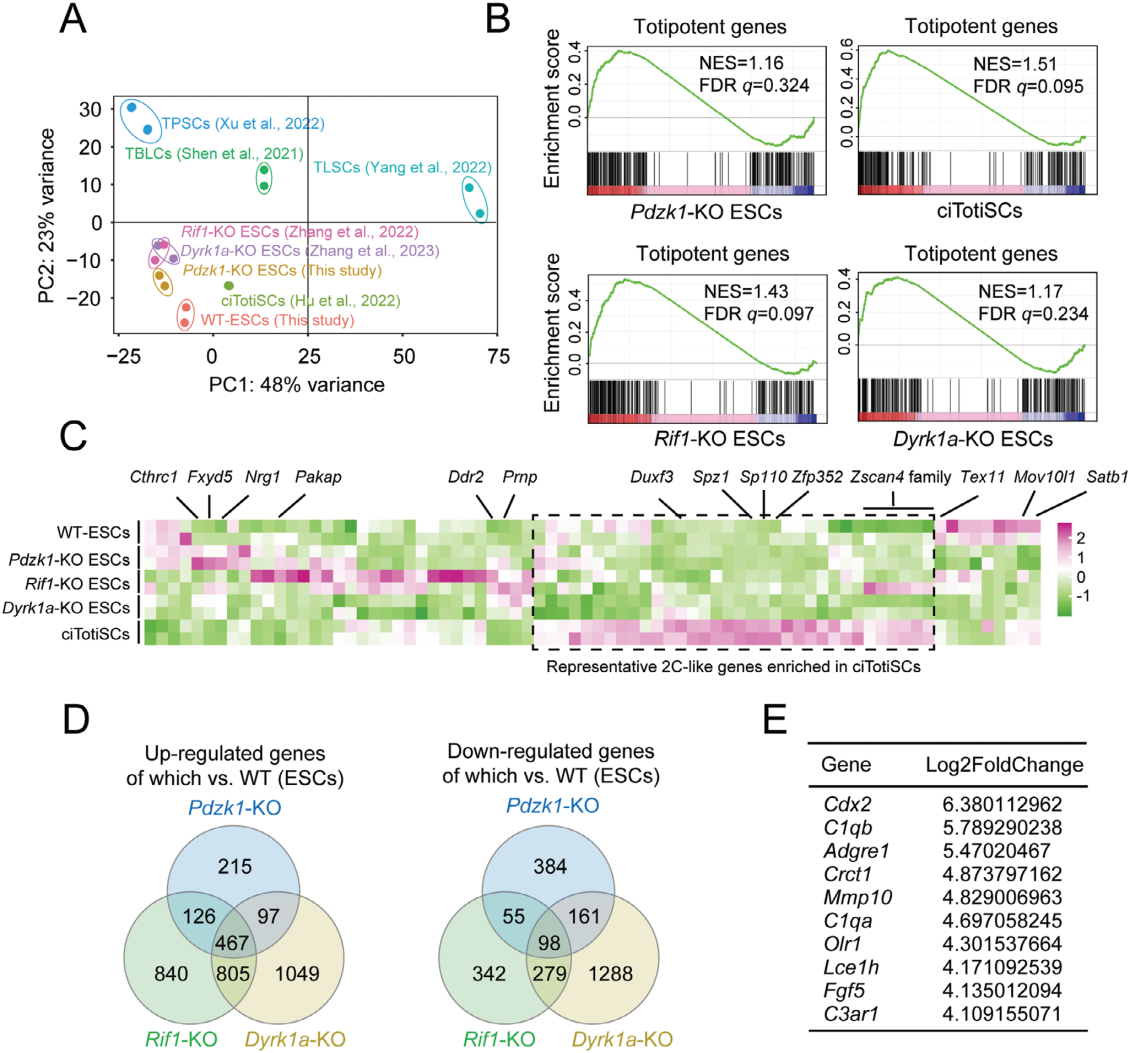
Bulk RNA‐seq analysis of *Pdzk1*‐KO ESCs. A) PCA of bulk RNA‐seq datasets of WT‐ESCs, *Pdzk1*‐KO ESCs, *Dyrk1a*‐KO ESCs, *Rif1*‐KO ESCs, TBLCs, TLSCs, TPSCs and ciTotiSCs. B) GSEA of DEGs associated with totipotent genes in *Pdzk1*‐KO ESCs, *Dyrk1a*‐KO ESCs, *Rif1*‐KO ESCs and ciTotiSCs. NES, normalized enrichment score; FDR, false discovery rate. C) Heatmap of representative 2C‐like genes in *Pdzk1*‐KO ESCs, *Dyrk1a*‐KO ESCs, *Rif1*‐KO ESCs, ciTotiSCs and WT‐ESCs. D) Venn diagram of the co‐upregulated and co‐downregulated DEGs among *Pdzk1*‐KO ESCs, *Dyrk1a*‐KO ESCs, and *Rif1*‐KO ESCs, each versus WT‐ESCs. E) Top 10 genes among the co‐upregulated DEGs among *Pdzk1*‐KO ESCs, *Dyrk1a*‐KO ESCs, and *Rif1*‐KO ESCs.

To further investigate why *Pdzk1*‐KO promoted the plasticity of ESCs, we analyzed the RNA‐seq results of *Pdzk1*‐KO ESCs. We compared our *Pdzk1*‐KO ESCs with the previously reported totipotent‐like stem cells in our group and other groups at the transcriptome level globally.^[^ ^16^, ^17^, ^20^, ^21^, ^22^, ^23^ ^]^ The PCA results revealed that *Pdzk1*‐KO ESCs were close to *Rif1*‐KO ESCs (our group), *Dyrk1a*‐KO ESCs (our group), and ciTotiSCs, being far from TBLCs, TPSCs, and TLSCs (**Figure** **5**A). Next, we detected whether *Pdzk1*‐KO activated a common 2‐cell‐like (2C‐like) specific reporter, MuERV‐L (MuERV‐L driving tdTomato), in cell cultures (normally 3.0–5.0% of WT‐ESCs). We found that the proportion of MuERV‐L+ cells in *Pdzk1*‐KO ESCs (11.0–14.8%) was greater than that in WT‐ESCs (3.5%) (Figure S5A,B, Supporting Information). To further assess the properties of *Pdzk1*‐KO ESCs, we performed gene set enrichment analysis (GSEA) and found that *Pdzk1*‐KO ESCs were also enriched in a previously reported totipotent gene set relative to WT‐ESCs,^[^ ^20^ ^]^ resembling those of ciTotiSCs, *Rif1*‐KO ESCs, and *Dyrk1a*‐KO ESCs. However, although *Pdzk1*‐KO ESCs were enriched in the totipotent gene set according to the GSEA results, their correlation and significance were still lower than those of ciTotiSCs (Figure 5B). Thus, we checked the expression levels of 2C‐like representative genes and other genes very carefully according to the heatmap results. Interestingly, some well‐known 2C‐like genes enriched in ciTotiSCs (including the *Zscan4* family, *Zfp352*, *Duxf3*), *Pdzk1*‐KO ESCs, *Rif1*‐KO ESCs, and *Dyrk1a*‐KO ESCs (three totipotent‐like stem cell lines in our group) did not significantly differ from those in WT‐ESCs, especially *Pdzk1‐KO* ESCs (Figure 5C). Compared with WT‐ESCs, ciTotiSCs exhibited significantly upregulated expression of these genes. We next sought to determine why *Pdzk1‐KO* ESCs exhibited totipotent‐like phenotypes without the activation of representative 2C‐like genes. Thus, we analyzed the differentially expressed genes (DEGs) of *Pdzk1*‐KO ESCs, *Rif1*‐KO ESCs, and *Dyrk1a*‐KO ESCs versus WT‐ESCs and found that the three groups had a total of 467 overlapping genes whose expression was upregulated and 98 overlapping genes whose expression was downregulated (Figure 5D). According to the GO analysis, the co‐upregulated genes were enriched mainly in the extracellular matrix and other pathways, whereas the downregulated genes were correlated mainly with carbohydrate catabolic processes and other pathways (Figure S5C, Supporting Information). The top ten genes among the co‐upregulated genes are shown and were used for further investigations (Figure 5E). However, whether these co‐upregulated genes are lineage transition activators warrants more evidence.

### 2.6 Activation of Lineage Inducers in Mouse ESCs can also Lead to a Totipotent‐Like State

**Figure 6:**
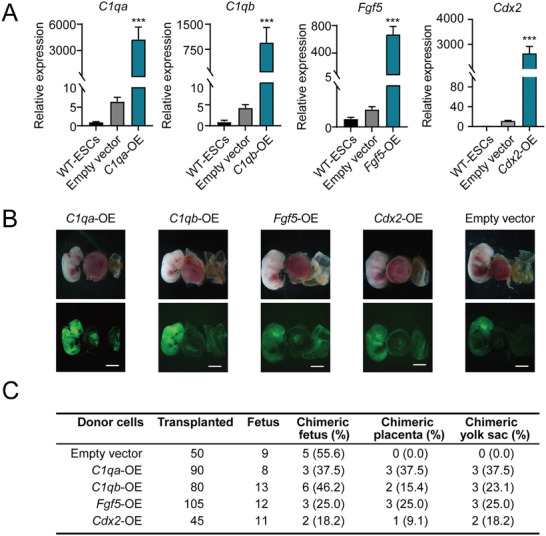
Validation of lineage inducers in the totipotent state. A) Expression levels of OE genes (*C1qa*, *C1qb*, *Fgf5*, and *Cdx2*) in OE‐ESCs, with the empty vector group and WT‐ESCs used as controls. The data are presented as the means ± SDs. ***p < 0.001. B) Images of E12.5 chimeras (including fetuses, placentas and yolk sacs) derived from GFP‐labeled *C1qa*‐OE, *C1qb*‐OE, *Fgf5*‐OE, and *Cdx2*‐OE ESCs, with the GFP‐labeled empty vector group used as a control. Scale bar, 5 mm. C) Summary of *C1qa*‐OE, *C1qb*‐OE, *Fgf5*‐OE, and *Cdx2*‐OE ESCs contributing to three lineages according to chimeric assays. D) Volcano plot of DEGs between *Pdzk1*‐KO ESCs and WT‐ESCs. *C1qa*, *C1qb*, *Fgf5*, and *Cdx2* are highlighted.

To address whether the overexpression (OE) of these upregulated genes in *Pdzk1*‐KO ESCs itself could trigger a totipotent‐like state, we randomly selected four of the top 10 genes among the genes co‐expressed with the genes mentioned above and overexpressed them individually in mouse ESCs via PB‐based vectors (Figure S6A, Supporting Information). The quantitative PCR (qPCR) results demonstrated that the *C1qa*‐OE, *C1qb*‐OE, *Fgf5*‐OE, and *Cdx2*‐OE subclones were obtained (**Figure** **6**A). To avoid unnecessary insertion mutations, we checked the insertion sites of PB in these subclones via inverse PCR. The results revealed that all the insertion sites were located in noncoding regions or harmless regions (Figure S6B,C, Supporting Information). Furthermore, we performed a chimera assay with these GFP‐labeled OE subclones separately, with an empty vector group used as a negative control. Surprisingly, bright‐field (BF) and FITC channel images of chimeric embryos and belongings at E12.5 demonstrated that all the tested OE subclones except those in the empty vector group could contribute to the fetus, placenta and yolk sac, suggesting that the activation of these lineage inducers could also increase the developmental potential (Figure 6B,C). Next, we performed qPCR and found that some TE marker (*Gata3*, *Tead4*, *Tfap2c* and *Cdx2*) and PrE marker (*Sox7*, *Foxa2*, *Gata6*, *Sox17*, and *Gata4*) genes were significantly upregulated in *C1qa*‐OE, *C1qb*‐OE, *Fgf5*‐OE and *Cdx2*‐OE ESCs compared with WT‐ESCs (Figure S6D,E, Supporting Information). In addition, we checked whether OE of exogenous *Cdx2* could activate endogenous *Cdx2* via qPCR. The results revealed that the expression level of endogenous *Cdx2* was significantly increased (Figure S6F, Supporting Information). All the above results might be the original force for cell fate alternation.

**Figure 7:**
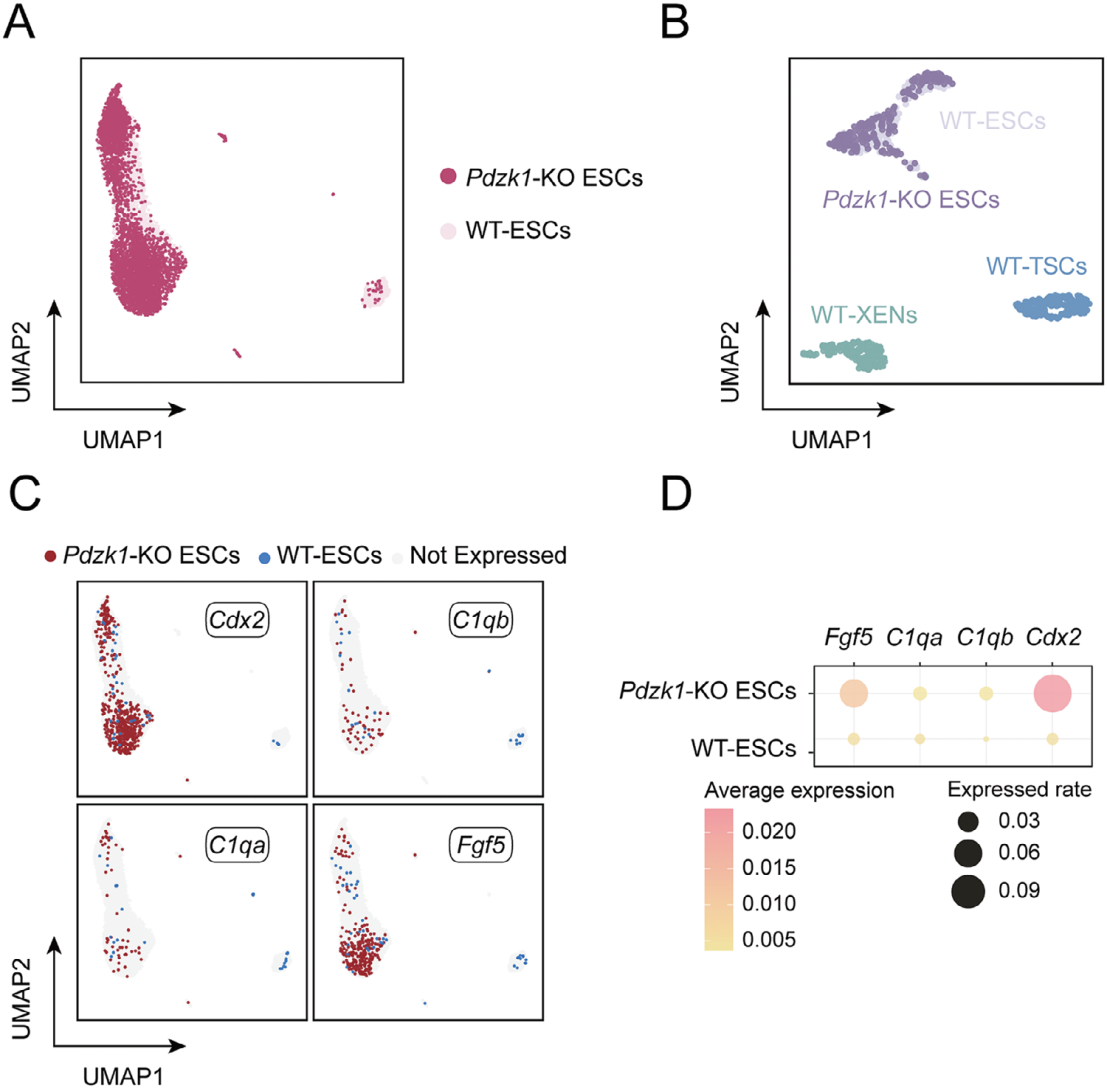
Single‐cell transcriptome profiles of *Pdzk1*‐KO ESCs and WT‐ESCs. A) UMAP plots of *Pdzk1*‐KO ESCs and WT‐ESCs. B) UMAP plots of *Pdzk1*‐KO ESCs, WT‐ESCs, WT‐TSCs, and WT‐XENs. C) Normalized expression of *C1qa*, *C1qb*, *Fgf5*, and *Cdx2* in *Pdzk1*‐KO ESCs and WT‐ESCs according to the UMAP at the single‐cell level. D) Dot plots indicating the expression of *C1qa*, *C1qb*, *Fgf5*, and *Cdx2* in *Pdzk1*‐KO ESCs and WT‐ESCs.

Next, we comprehensively investigated the bioinformatics data. These newly identified lineage inducers (*C1qa*, *C1qb*, *Fgf5*, and *Cdx2*) might explain the increase in totipotency in *Pdzk1*‐KO ESCs, as highlighted in the volcano plot of DEGs between *Pdzk1*‐KO ESCs and WT‐ESCs (Figure S7A, Supporting Information). To confirm the properties of *Pdzk1*‐KO ESCs, we compared them with WT‐ESCs, and the results indicated their similarity (**Figure** **7**A). To further confirm the homogeneity of *Pdzk1*‐KO ESCs, we compared them with WT‐ESCs, WT‐TSCs, and WT‐XENs via a UMAP plot. The results revealed that *Pdzk1*‐KO ESCs were still ESCs, in place of WT‐TSCs or WT‐XENs (Figure 7B), suggesting that the depletion of *Pdzk1* induced a totipotent‐like state rather than resulting in a mixed cell population primed for different lineages. Single‐cell RNA‐seq analysis further confirmed that the proportion of *Pdzk1*‐KO ESCs expressing these lineage inducers was greater than that of WT‐ESCs (Figure 7C; Figure S7B, Supporting Information). The expression levels of corresponding genes in WT‐ESCs and *Pdzk1*‐KO ESCs indicated that the expression levels of specific genes (including *Cdx2*, *C1qa*, *C1qb*, and *Fgf5*) in *Pdzk1*‐KO ESCs are higher than those in WT‐ESCs (Figure 7D). In total, *Pdzk1*‐KO activated several key lineage inducers to possess lineage transition potential and promote a totipotent‐like state.

## 3 Discussion

Zygotes and early blastomeres have the ability to form entire organisms and extraembryonic counterparts, respectively, representing the highest level of developmental potential, and are thereby termed totipotent cells. Although 2C‐like cells in ESCs exhibit developmental potential in the formation of extraembryonic tissues, it is controversial whether it is necessary to express these well‐known 2C‐like‐specific genes to gain totipotent‐like phenotypes. In this study, we found that the deletion of *Pdzk1* facilitates the development of a more plastic state in mouse ESCs, which possesses embryonic and extraembryonic developmental potential both in vitro and in vivo (Figures 1 and 2). Moreover, *Pdzk1*‐KO ESCs self‐organized to form blastoids with high formation efficiency (Figure 3A,B). The *Pdzk1*‐KO blastoids resembled cells from E4.5 embryos at the single‐cell level (Figure 4D), contained three lineages of blastocysts, and were capable of establishing subsequent stem cell lines (Figure 3E–H). They also have the ability to further develop to the peri‐implantation stage in an IVC system (Figure 3I–K) and to induce decidualization after implantation in vivo (Figure S3C–F, Supporting Information). Taken together, these findings indicate that *Pdzk1*‐KO enables ESCs to exhibit a very strong totipotent‐like state. However, *Pdzk1*‐KO ESCs do not exhibit significantly higher expression levels of some well‐known 2C‐like marker genes (including *Dux*, *Spz1*, *Zscan4* family genes)^[^ ^38^, ^39^ ^]^ than WT‐ESCs do (Figure 5C). To this end, these well‐known 2C‐like genes are not essential for activating totipotent‐like phenotypes in ESCs.

In some recent works, mouse totipotent stem cells were successfully isolated and stably maintained via the inhibition of spliceosome activity (TBLCs)^[^ ^20^ ^]^ or epigenetic modulation (TLSCs, TPSCs, and ciTotiSCs).^[^ ^21^, ^22^, ^23^ ^]^ These cells demonstrate extensive activation of 2C‐like genes at the molecular level and can contribute to embryonic and extraembryonic tissues. However, some inadequacies remain. For example, the proliferation ability of TBLCs is restricted compared with that of pluripotent ESCs, which lack long‐term culture capability. However, the deletion of *Pdzk1* did not affect the proliferation rate or viability of ESCs (Figure S1, Supporting Information). All the above‐reported totipotent‐like stem cells cannot be passaged more than 25 times, although they claim that their stem cells maintain totipotency beyond pluripotency, whereas *Pdzk1*‐KO ESCs show totipotent‐like potential regardless of the number of passages. Interestingly, according to our data, *Pdzk1*‐KO ESCs are more consistently correlated with 32‐cell mouse embryos than with 2‐cell embryos (Figure 4E,F). Thereafter, we checked three totipotent‐like stem cell lines in our group (Figure 5A–C) at the global transcriptome level and found that several overlapping coupregulated gene sets were not related to totipotent‐like phenotypes (Figure 5D,E). After a round of careful validation experiments, a few lineage inducers (*C1qa*, *C1qb*, *Fgf5*, and *Cdx2*) were found to be essential for breaking down lineage barriers and activating a totipotent‐like state in *Pdzk1*‐KO ESCs (Figure 6). The Na‐H exchanger NHE3 and the Cl‐ anion exchanger CFEX (SLC26A6, PAT1) play principal roles in the reabsorption of Na and Cl in the proximal tubule of the mammalian kidney. A previous study reported that NHE3 and CFEX directly interact with PDZK1 and that this interaction is mediated through their C‐terminal PDZ interaction sites.^[^ ^40^ ^]^ Interestingly, cellular mechanical properties, including the lineage commitments of stem cells, play important roles in multiple cell fate determination events.^[^ ^41^, ^42^ ^]^ Whether the increase in totipotency of *Pdzk1*‐KO ESCs is correlated with the loss of Na+/H+ exchange factors requires further investigation. To explore whether *Pdzk1* is correlated with entry or exit from the totipotent state during mouse preimplantation development, we first checked the expression patterns of *Pdzk1* in WT mouse embryos. We found that the expression level of *Pdzk1* in zygotes was highest during early embryonic development. When the embryos develop to the 2‐cell stage, the expression level of *Pdzk1* decreases to nearly zero immediately (Figure S7C, Supporting Information). These findings indicate that the disappearance of *Pdzk1* may be a key point at which the 2‐cell stage activates transcription of the zygotic genome. The hippo effectors YAP/TAZ act as on‐off mechanosensing switches by sensing modifications in the extracellular matrix (ECM) composition and mechanics.^[^ ^43^, ^44^ ^]^ In embryos, YAP1 is localized to the nuclei of outside cells, whereas in inside cells beginning in morula‐stage embryos, YAP1 is phosphorylated in the cytoplasm. The modulation of YAP1 activity leads to changes in *Cdx2* expression, which is important for lineage specification.^[^ ^45^, ^46^ ^]^ To determine the probable reason for the increase in totipotency in *Pdzk1*‐KO ESCs, we determined the location of YAP1 in *Pdzk1*‐KO ESCs and WT‐ESCs. The results revealed that YAP1 is located in the nucleus in *Pdzk1*‐KO ESCs along with CDX2, TFAP2C, and EOMES, which is completely different from WT‐ESCs (Figure S7D–F, Supporting Information). This may explain why the deletion of *Pdzk1* promotes extraembryonic developmental potential and results in a totipotent‐like status.

Overall, our findings demonstrate that capturing totipotency can be achieved just by deleting one single gene (*Pdzk1*‐KO) in mouse ESCs, notably a gene that has never been studied for its developmental potential. The *Pdzk1*‐KO ESCs fulfill several key criteria for a useful model of totipotent‐like phenotypes: 1) they are tripotent to ESCs, XENs, and TSCs in vitro; (2) they contribute to the embryonic fetus, extraembryonic yolk sac and placenta according to chimera assays; and (3) they self‐organize to form blastocyst‐like structures, with pre‐ and peri‐implantation development potential. The *Pdzk1*‐KO blastoids not only exhibited high formation efficiency but also clustered the scRNA‐seq data into three unambiguous lineages. Thus, this study provides a valuable model for both reproductive and development research via a scalable and well‐controlled strategy, offering a unique platform to study embryogenesis, early developmental defects, and other developmental concerns. Finally, this study provides novel insight that the phenotypes of the totipotent‐like state can be acquired through the activation of some lineage inducers.

## 4 Experimental Section

### Mice

All mice were generated and housed at the Nankai animal resources center under specific pathogen‐free (SPF) conditions. All the animal experiments complied with the guidelines of the Institutional Animal Care and Use Committee of Nankai University.

### Cell Culture for ESCs and TSCs

All the ESC lines were cultured on feeder cells in t2i/L ESC medium^[^ ^47^ ^]^ with slight modifications. All types of TSCs in this study were cultured on feeder cells in mouse TSC medium published recently.^[^ ^48^ ^]^ Briefly, TSC medium consisted of 30% TSC basic medium, 70% MEF‐conditioned medium (70CM) supplemented with 25 ng ml^−1^ human recombinant FGF4 (MCE, HYP7014) and 1 µg ml^−1^ heparin (MCE, HY‐17567A). All the cells in this study were cultured in humidified incubators at 37 °C in 5% CO_2_ and were routinely checked for mycoplasma via PCR.

### Plasmid Construction and Electroporation

For the KO of *Pdzk1*, two sgRNAs were designed according to CRISPOR (www.crispor.tefor.net) and cloned and inserted into a PX458 plasmid (Addgene, #48138). Approximately 8 µg of plasmid was electroporated into 1 × 10^6^ ESCs via an electroporator (NEON, Invitrogen) at 1400 V for 10 ms with 3 pulses. To label the ESCs with a β‐actin‐GFP reporter, the β‐actin‐GFP donor vector and two β‐actin sgRNAs reported previously were used.^[^ ^16^ ^]^ To construct the overexpression plasmids, the coding sequences (CDSs) of *C1qa*, *C1qb*, *Fgf5*, or *Cdx2* were ligated to a modified *piggyBac* vector (SBI, PB513B‐1) as indicated (Figure 6A). The PBase plasmid (SBI, PB210PA‐1) was purchased from a local agent. All the primers used are listed in Table S1 (Supporting Information).

### Generation of iTSCs and iXENs

To induce TSCs, ≈1 × 10^5^ *Pdzk1*‐KO ESCs or WT‐ESCs were seeded into gelatin (Sigma, V900863) precoated in dishes with TSC medium (70 CM/F4H).^[^ ^4^ ^]^ After 14 days, CDCP1‐positive cells from the cell cultures were sorted using a flow cytometer (Beckman Coulter, MoFlo Astrios EQ) with a CDCP1 antibody (R&D, AF4515). These sorted cells were subsequently expanded in TSC medium to derive iTSCs.

For the induction of XENs, ≈1 × 10^6^ *Pdzk1*‐KO ESCs and WT‐ESCs were seeded into gelatin precoated dishes and cultured in iXEN medium. The iXEN medium contained RPMI 1640 (Thermo, 61870036) supplemented with 15% FBS (VivaCell, c04002‐500), 0.1 mm β‐mercaptoethanol (Thermo, 21985023), 0.01 µm retinoic acid (MCE, HY‐14649), 10 ng ml^−1^ activin A (MCE, HY‐P700159AF), 24 ng ml^−1^ bFGF (Peprotech, 100–18B) and 1 µg ml^−1^ heparin (MCE, HY‐17567A). After 2 days, all the differentiated cells were passaged and plated into MEF precoated dishes and cultured in standard XEN medium. XEN medium contained RPMI 1640 (Thermo, 61870036) supplemented with 15% FBS and 0.1 mm β‐mercaptoethanol. Then, XEN‐like cells were picked, placed in MEF precoated or gelatin precoated dishes and cultured in XEN medium.

For immunofluorescence staining, cells or zona pellucida‐removed embryos were fixed with 4% paraformaldehyde (Sigma, 158127) at room temperature (RT) for 30 min. After blocking and permeabilization with 2% BSA (Sigma, V900933) and 0.3% Triton X‐100 (Sigma, T8787, diluted in PBS) at RT for 2 h (for lVC Day 4 blastoids, 2% BSA and 1% Triton X‐100 were used), the samples were incubated with primary antibodies at 4 °C overnight and with secondary antibodies at RT for 1 hour. The nuclei were stained with Hoechst 33342 (Thermo, H3570). F‐actin was stained with phalloidin (MEC, HY‐D1816). The primary antibodies used were CDX2 (BioGenes, MU392A), OCT4 (Abcam, ab181557), GATA6 (CST, 5851), EOMES (Abcam, ab23345), TFAP2C (Santa Cruz, sc‐12762), and YAP1 (ABclonal, A1002). The secondary antibodies used included FITC‐conjugated goat anti‐rabbit IgG (H+L) (ABclonal, AS011) and FITC‐conjugated goat anti‐mouse IgG (H+L) (ABclonal, AS001). The samples were imaged via a confocal laser scanning microscope (Leica, TCS SP8).

### Karyotype Analysis and Teratoma Formation

For karyotype analysis, the cells were incubated with 0.2 µg mL^−1^ nocodazole (MCE, HY‐13520) for 8 hours. The samples were treated with a hypotonic solution (methanol (Sigma, 34860): acetic acid (Sigma, 71251) = 3:1) for 20 min twice, dropped onto ice‐precooled slides, and stained with Giemsa (Sigma, 32884) for 5 min before observation. Images were captured via a confocal laser scanning microscope (Leica, TCS SP8).

For teratoma analysis, 100 µl of *Pdzk1*‐KO ESCs or WT‐ESCs suspended (1 × 10^7^ cells) in PBS were injected subcutaneously into the limbs of 8‐week‐old male severe combined immunodeficiency (SCID) mice. Teratomas were dissected 3 weeks later and fixed in 4% paraformaldehyde for paraffin sectioning, H&E staining and further analysis.

### Mouse Chimeric Assay

To obtain embryos, hormone‐treated adult female mice were mated with adult male mice. Two‐cell‐stage embryos were flushed out and collected at E1.5. All embryos were cultured in KSOM medium (Millipore, MR‐020P‐5F) in humidified incubators at 37 °C in 5% CO_2_ until subsequent micromanipulation.

To construct chimeric embryos, GFP‐labeled ESCs were injected into 4–8‐cell‐stage embryos to construct chimeric embryos (8–10 donor cells per embryo). The reconstructed chimeric embryos were cultured in KSOM for another day to form blastocysts. Next, image capture, fixation, and immunofluorescence staining were performed. In parallel, the reconstructed chimeric embryos were transferred to the uteri of pseudopregnant mice. The conceptuses were dissected at E12.5 for further image capture, flow cytometric analysis, immunohistochemistry, and bulk RNA‐seq.

### Immuno‐Fluorescent Sections

E12.5 placentas were embedded in O.C.T. (SAKURA, 4583) compound and frozen at −80 °C. Tissue sections (8 mm thick) were cut with a cryostat microtome and transferred onto histological slides. The samples were permeabilized and blocked with 1% Triton X‐100 and 5% BSA at room temperature for 1 hour. Next, the samples were incubated with diluted primary antibodies overnight at 4 °C. The primary antibodies used were PROLIFERIN (Santa Cruz, sc‐271891), GCM1 (Santa Cruz, sc‐101173), TPBPA (Abcam, ab320823), and TFAP2C. Following three washes in PBS, the samples were incubated with diluted secondary antibodies at room temperature for 1 h. After washing three times in PBS, the nuclei were stained with Hoechst 33342. The samples were imaged via total internal reflection fluorescence (TIRF) microscopy (Leica, DMi8).

### Generation of Blastoids

Blastoids were generated as described previously.^[^ ^29^ ^]^ Briefly, ≈500 *Pdzk1*‐KO and WT‐ESC cells were seeded into one well of a 96‐well ultralow attachment plate (Corning, 7007) and cultured in blastoid medium, which was composed of 25% TSC medium, 25% N2B27 basal medium, 50% KSOM medium supplemented with 2 µm Y‐27632 (Selleck, S1049), 12.5 ng mL^−1^ FGF4 (Peprotech, 10031), 0.5 µg mL^−1^ heparin (MCE, HY17567A), 3 µm CHIR99021 (MCE, HY‐10182), 5 ng mL^−1^ BMP4 (MCE, HYP7007), and 0.5 µm A83‐01 (MCE, HY‐10432). After 8 days, the blastoids were manually collected via a mouth pipette under a stereomicroscope (Nikon, Ti‐S) for subsequent experiments.

### Derivation of BESCs, BTSCs, and BXEN Cells

GFP‐labeled blastoids were used for cell line derivation. For derivation of BESCs and BTSCs, blastoids were plated in one well of a 24‐well TC plate (NEST, 702001) with feeder cells in t2i/L medium (mentioned above) or TSC medium. The outgrowths were dissociated and plated into fresh feeder cells and fresh media. Typical ES cell colonies and TS colonies could be observed and passaged. The cell line derivations were performed.

BXEN cells were derived following a previously reported protocol with slight modifications.^[^ ^49^ ^]^ Briefly, blastoids were plated in gelatin precoated wells with XEN medium. The outgrowths were dissociated and further cultured in the XEN medium mentioned above.

### In Vitro Culture of Blastoids

*Pdzk1*‐KO blastoids were cultured to the postimplantation stage via a protocol previously described for mouse blastocysts in vitro.^[^ ^27^ ^]^ Briefly, *Pdzk1*‐KO blastoids were washed twice with M2 medium (Sigma, M7167) and transferred into an ibiTreat µ‐plate well (ibidi, 80826) containing IVC1 medium. Once the *Pdzk1*‐KO blastoids attached (usually 2 days after plating), the medium was changed to IVC2 medium for another 2 days of culture. IVC1 medium contained DMEM/F12 (Thermo, 11320033) supplemented with 20% FBS, 2 mM GlutaMAX (Thermo, 35050061), 1% penicillin‒streptomycin, 1×ITS‐X (Thermo, 12634020), 8 nm β‐mercaptoethanol (MCE, HYB0141), 200 ng ml^−1^ progesterone (MCE, HYN0437) and 25 µm N‐acetyl‐l‐cysteine (MCE, HY‐B0215). IVC2 medium contained DMEM/F12 supplemented with 30% KOSR (Thermo, 10828028), 2 mm GlutaMAX, 1% penicillin‒streptomycin, 1×ITS‐X, 8 nm β‐mercaptoethanol, 200 ng ml^−1^ progesterone, and 25 µm N‐acetyl‐l‐cysteine.

### Analysis of Bulk RNA‐seq

All the raw data were generated via PE150. The clean data were mapped to the mm10 reference genome. STAR 2.5.3 was used for paired‐end clean read alignment, and HTseq was used for read count calculation and output. The DESeq2 R package was used for DEG analysis. Genes with expression fold changes >2 and adjusted P values <0.05 were retained for Gene Ontology (GO) analysis and volcano plot generation. GSEA (v4.2.4) was used to determine whether the set of 2C‐like and totipotent genes was significantly enriched in *Pdzk1*‐KO ESCs compared with WT‐ESCs on the basis of the RNA‐seq data. The normalized enrichment score (NES) and FDR q value are indicated for the gene set. The data used in this study were obtained from previous publications: *Rif1*‐KO ESCs: GSE189683; *Dyrk1a*‐KO ESCs: CRA011851; TPSCs: GSE183522; TLSCs: GSE166216; TBLCs: GSE168728; and ciTotiSCs: GSE1885005.

### Analysis of 10× Genomics Single‐Cell RNA‐seq

The cells were washed and resuspended in 1× PBS (calcium‐ and magnesium‐free) supplemented with 10% FBS. The cell suspension was loaded onto a Chromium single‐cell controller (10× Genomics). The Chromium Single Cell 3ʹ GEM, Library and Gel Bead Kit v3.1 (10× Genomics, 1000075) and Chromium Single Cell B Chip Kit (10× Genomics, 1000074) were used to generate single‐cell gel beads in the emulsion according to the manufacturer's protocol. Reverse transcription was performed on a Mastercycler nexus gradient (Eppendorf) at 53 °C for 45 min, followed by incubation at 85 °C for 5 min and holding at 4 °C. Single‐cell RNA‐seq libraries were constructed via the single‐cell 3ʹ library and Gel Bead Kit V3.1 according to the manufacturer's instructions. The libraries were finally sequenced via an Illumina NovaSeq 6000 sequencer with a sequencing depth of at least 100 000 reads per cell with a paired‐end 150 bp (PE150) reading strategy (performed by Gene Denovo, Guangzhou).

The single‐cell RNA‐seq data were collected and mapped to the mouse reference genome mm10 via Cell Ranger 7.1.0 for all the samples. Preprocessing, data normalization, batch correction, clustering, and visualization of single‐cell datasets were performed via Seurat 4.4.0^[^ ^36^ ^]^ in R environment version 4.3.2. The data used in this study were obtained from previous publications: blastocyst: GSE145609;^[^ ^34^ ^]^ GSE100597;^[^ ^35^ ^]^ GSE135701;^[^ ^29^ ^]^ zygote: 2‐cell, 4‐cell, late 4‐cell, 8‐cell, 16‐cell, 32‐cell: GSE136714;^[^ ^37^ ^]^ E3.5, E4.5, E5.5, E6.5, E6.75: GSE100597;^[^ ^35^ ^]^ TBLCs: GSE168728;^[^ ^20^ ^]^ D. EPSCs, L. EPSCs: GSE145609;^[^ ^34^ ^]^ and ciTotiSCs: GSE185001.^[^ ^22^ ^]^ In detail, quality control of *Pdzk1*‐KO ESCs and WT‐ESCs was performed to remove the cells with (1) total UMI counts <2000, (2) detected gene number <1500, or (3) mitochondrial UMI counts >20%, and quality control of *Pdzk1*‐KO blastoids was performed to retain the cells with (1) a detected gene number between 200 and 9000 and (2) mitochondrial UMI counts <5%.

For the comparison between *Pdzk1*‐KO ESCs and WT‐ESCs, the UMI counts were normalized with the normalizeData function using the default settings; then, datasets were used to select the 2000 most variable genes with the FindVariableFeatures function and scaled with the ScaleData function before computing PCA with the RunPCA function. Considering the large dataset, integration was performed with the RunHarmony function from the R package Harmony 1.2.0^[^ ^50^ ^]^ and the RunUMAP function with the first 20 PCs was used to perform nonlinear dimension reduction and clustering with a resolution setting of 0.1. The DEGs were analyzed via the FindAllMarkers function to identify 2 cell‐like cells.

For the comparison between *Pdzk1*‐KO blastoids and blastocysts, normalization and variance stabilization of the datasets were performed via the SCTransform function with default settings. For integration, the 2000 most variable genes were identified via the SelectIntegrationFeatures function, and integration anchors were identified on the basis of these genes via the PrepSCTIntegration function and the FindIntegrationAnchors function with normalization.method = “SCT” and k.filter = 50. The data were subsequently integrated via the IntegrateData function with normalization.method = “SCT” and k.weight = 50. The pipeline was performed with integrated datasets via the following functions: RunPCA, RunUMAP with the first 20 PCs, FindNeighbors with the first 20 PCs, and FindClusters with a resolution of 0.4. Annotated cell information^[^ ^34^ ^]^ was collected from three lineages, EPI, TE, and PrE, of blastocysts and used singleR 2.4.1^[^ ^51^ ^]^ to complete the automated annotation of clusters on the basis of a self‐constructed database.

For the comparison based on the stages of embryonic development and other stem cells, scMerge 1.18.0^[^ ^52^ ^]^ was used to merge and normalize the single‐cell RNA‐Seq datasets to remove unwanted variation with the standard semisupervised scMerge2 pipeline. The adjusted matrix was returned and aggregated into one “pseudobulk” sample per label with the AverageExpression function from the Seurat 4.4.0 package. The hierarchical clustering tree was constructed via the BuildClusterTree function, PCA was performed via the prcomp function, and visualization was performed via ggplot2 3.5.0.

## Conflict of Interest

The authors declare no conflict of interest.

## Author Contributions

W.Z., Y.Z., Z.Y., J.Y., and H.W. contributed equally to this work. L.S. and Q.G. designed and supervised the study. W.Z., Y.Z., Z.Y., and H.W. performed most of the experiments. W.Z., J.Y., and X.Z. analyzed the bioinformatic data. S.N., X.L., and Q.J. participated in part of the cell culture and molecular experiments. W.Z., D.D., C.T., X.Z., Q.G., and L.S. wrote the manuscript draft. Q.G. and L.S. confirmed the final manuscript.

## Supporting information



Supporting Information

## Data Availability

The data underlying this article are available in the article and in its online supplementary material. The RNA‐seq and 10× Genomics raw dataset in this study have been deposited in the Genome Sequence Archive of the Beijing Institute of Genomics (BIG) Data Center with accession numbers CRA020593.
