# Supplementary material for: Capture of Totipotency in Mouse Embryonic Stem Cells in the Absence of Pdzk1
Source: Adv Sci (Weinh). 2024 Dec 4;12(6):2408852. doi: 10.1002/advs.202408852 (PMC11809344; doi:10.1002/advs.202408852)
Supplement: Supplementary file 1 — Supporting Information [file ADVS-12-2408852-s001.docx]

**Supplementary Information**

**Title:** Capture of totipotency in mouse embryonic stem cells in the absence of *Pdzk1*

**Running title:** A path to totipotency in ESCs

**Authors and affiliations:**

*Wenhao Zhang*, *Yiding Zhao*, *Zhe Yang*, *Jing Yan*, *Haisong Wang*, *Shaochen Nie*, *Qingshen Jia*, *Dan Ding*, *Chao Tong**, *Xiao-Ou Zhang**, *Qian Gao** and *Ling Shuai**

W. Zhang, Y. Zhao, Z. Yang, S. Nie, Q. Jia, D. Ding, Q. Gao*, L. Shuai*

State Key Laboratory of Medicinal Chemical Biology and College of Pharmacy and Nankai Animal Resources Center and Reproductive Regulation and Institute of Transplantation Medicine

Nankai University

Tianjin 300350, China.

Email: gaoqian@nankai.edu.cn (Q.G.); lshuai@nankai.edu.cn (L.S.)

Q. Gao*

Tianjin Key Laboratory of Animal and Plant Resistance, College of Life Sciences

Tianjin Normal University

Tianjin 300387, China.

Email: gaoqian@nankai.edu.cn (Q.G.)

J. Yan, X. Zhang*

Shanghai Key Laboratory of Maternal and Fetal Medicine, Clinical and Translational Research Center of Shanghai First Maternity and Infant Hospital, Frontier Science Center for Stem Cell Research, School of Life and Science and Technology

Tongji University

Shanghai 200092, China.

Email: zhangxiaoou@tongji.edu.cn (X.Z.)

H. Wang

Reproductive Medical Center, Henan Key Laboratory of Reproduction and Genetics

The First Affiliated Hospital of Zhengzhou University

Henan 450052, China.

C. Tong*

National Clinical Research Center for Child Health and Disorders,

Ministry of Education Key Laboratory of Child Development and Disorders,

Children’s Hospital of Chongqing Medical University

Chongqing 401122, China

Email: chaotong@hospital.cqmu.edu.cn (C.T.)


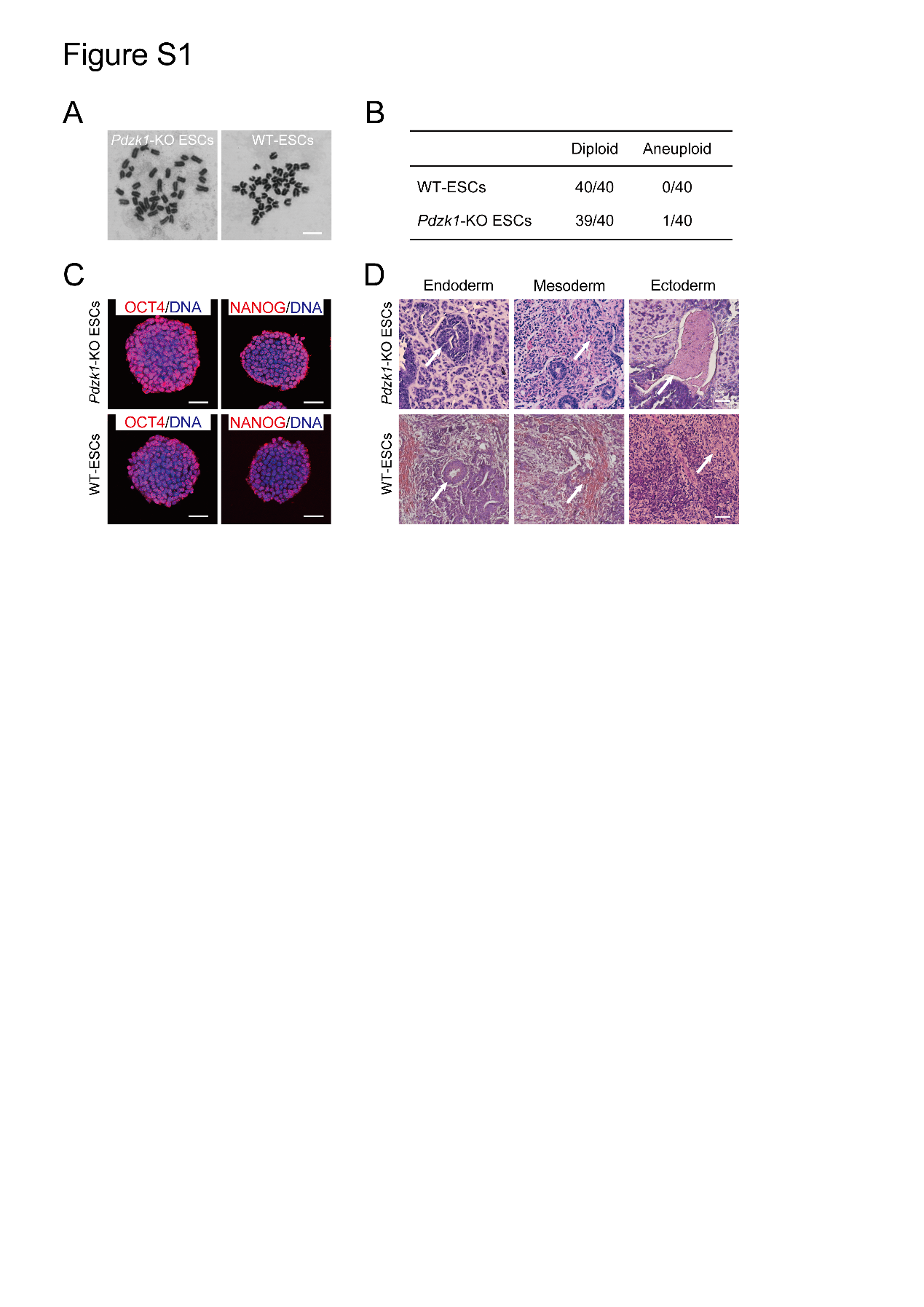


**Figure S1. Karyotype and pluripotency analysis of *Pdzk1*-KO ESCs**

1. Chromosome spread analysis of *Pdzk1*-KO ESCs and WT-ESCs. Scale bar, 10 μm.
2. Statistical analysis of chromosome numbers of *Pdzk1*-KO ESCs and WT-ESCs.
3. Immunofluorescence of ESC-specific markers (OCT4 and NANOG) in *Pdzk1*-KO ESCs and WT-ESCs. DNA is stained with Hoechst 33342 (blue). Scale bar, 50 μm.
4. Teratomas formed from *Pdzk1*-KO ESCs and WT-ESCs are identified by hematoxylin and eosin (H&E) staining. Scale bars, 100 μm. The shown tissues are glands (endoderm), blood (mesoderm) and neural tissue (ectoderm) as white arrow indicating, respectively.


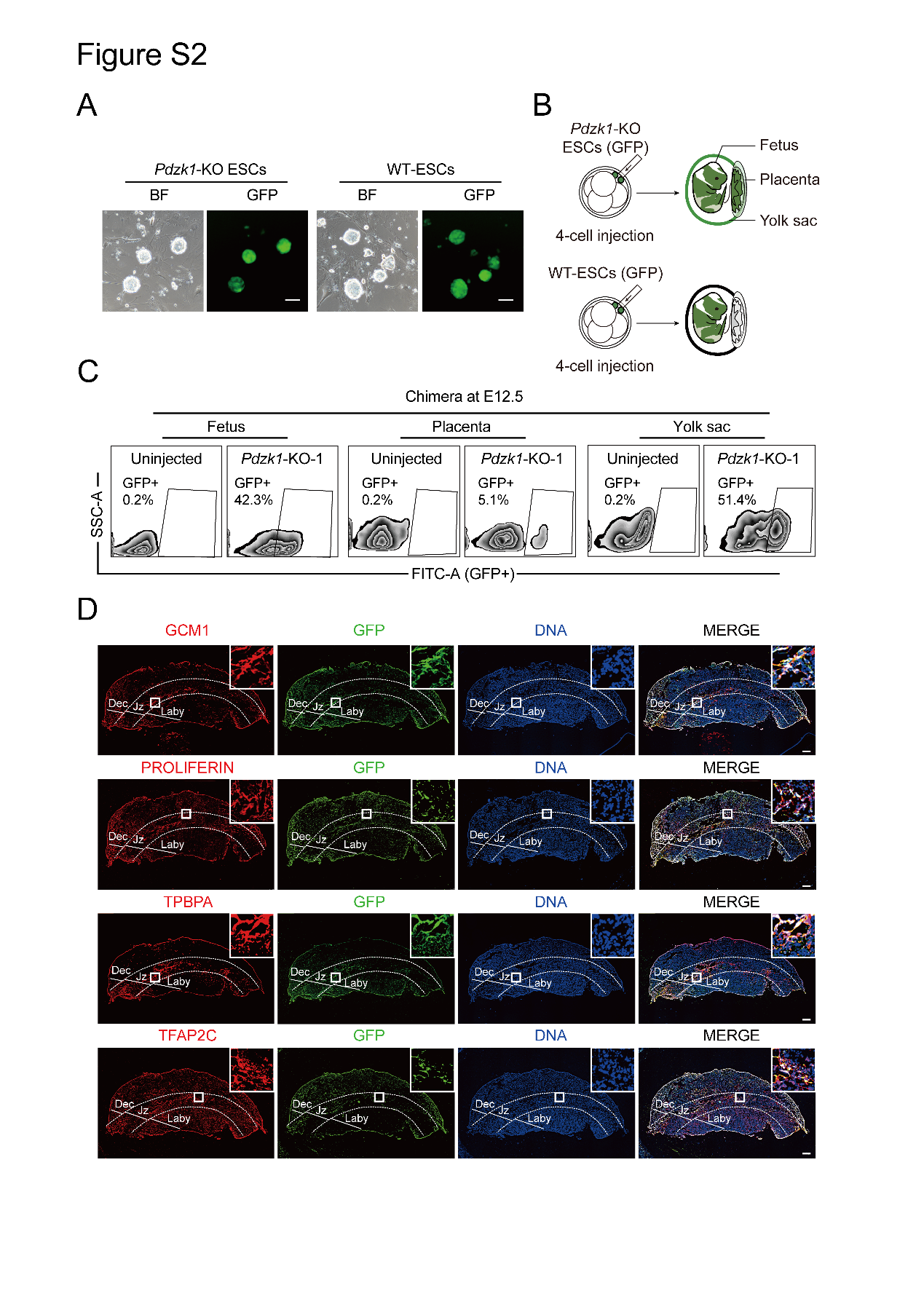


**Figure S2. Contribution of *Pdzk1*-KO ESCs to embryonic and extraembryonic tissues at E12.5**

1. Images of GFP-labeled *Pdzk1*-KO ESCs and GFP-labeled WT-ESCs (in bright field (BF) and in FITC channels). Scale bar, 100 μm.
2. Schematic overview of the developmental contribution of microinjected GFP-labeled *Pdzk1*-KO ESCs and GFP-labeled WT-ESCs in E12.5 chimeras.
3. FACS analysis of GFP+ cells in fetus, placenta and yolk sac in E12.5 chimeras derived from GFP-labeled *Pdzk1*-KO ESCs. The percentages of GFP+ cells in fetus, placenta and yolk sac from *Pdzk1*-KO chimera are 42.3%, 5.1% and 51.4%, respectively, with uninjected ones (fetus, placenta and yolk sac) at E12.5 as negative controls.
4. Immunofluorescence staining of placental-specific markers (GCM1, PROLIFERIN, TPBPA and TFAP2C, each in red) in the GFP-labeled *Pdzk1*-KO ESC-derived E12.5 chimeric placentas. The white rectangles indicate that GFP+ cells contribute to the tissues expressing specific markers, respectively. Scale bar, 500 μm.


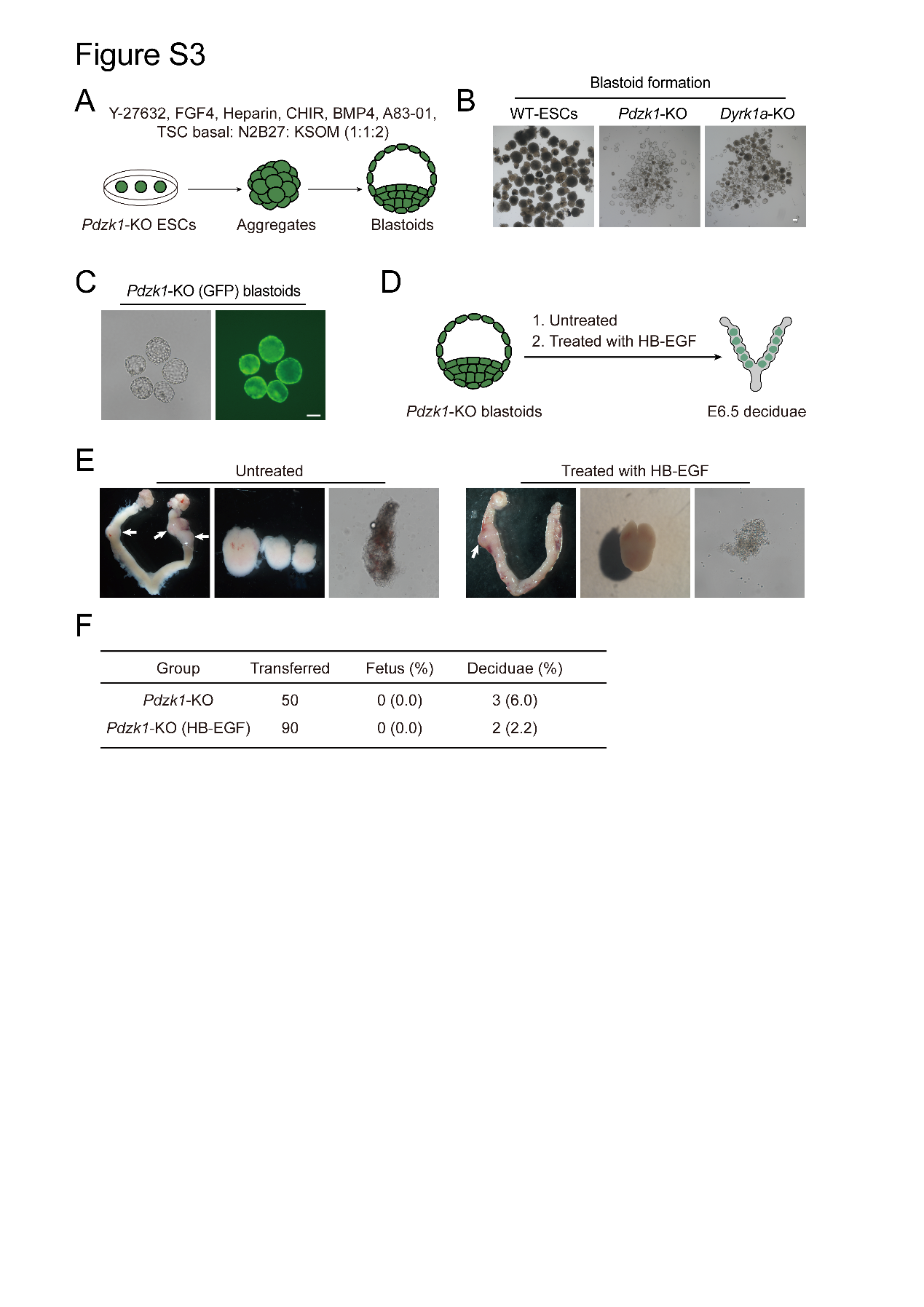


**Figure S3. *In vivo* development of** ***Pdzk1*-KO blastoids in uterus**

1. Schematic overview of *Pdzk1*-KO blastoids formation procedure *in vitro*.
2. The images of *Pdzk1*-KO blastoids, *Dyrk1a*-KO blastoids and the aggregating spheres derived from WT-ESCs as a control in BF channel. Scale bar, 100 μm.
3. Images of blastoids derived from GFP-labeled *Pdzk1*-KO ESCs (in BF and in FITC channels). Scale bar, 100 μm.
4. Schematic overview of *Pdzk1*-KO blastoids transfer and subsequent development assessment.
5. The images showing the formation of deciduae in the mouse uterus at E6.5 after *Pdzk1*-KO blastoids transfer as white arrow indicating (within and without HB-EGF groups).
6. Summary of formed deciduae derived from *Pdzk1*-KO blastoids (within and without HB-EGF) at E6.5.


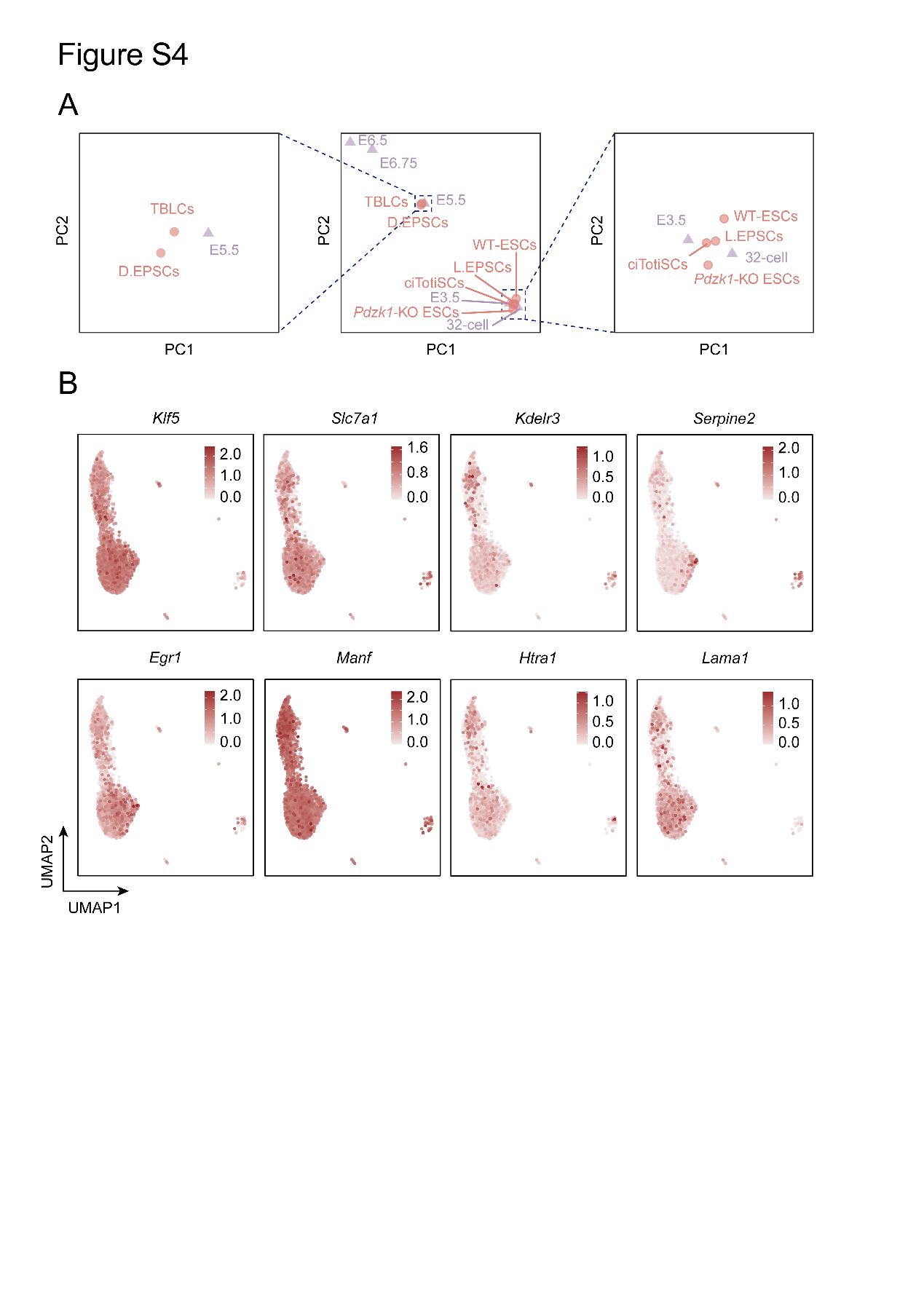


**Figure S4. Single-cell transcriptome analysis of *Pdzk1*-KO ESCs**

1. PCA plot of *Pdzk1*-KO ESCs, WT-ESCs, ciTotiSCs, TBLCs, L. EPSCs, D. EPSCs and mouse embryos at different stages at the single-cell level.
2. The expression patterns of representative 32-cell embryo marker genes in *Pdzk1*-KO ESCs at the single-cell level.


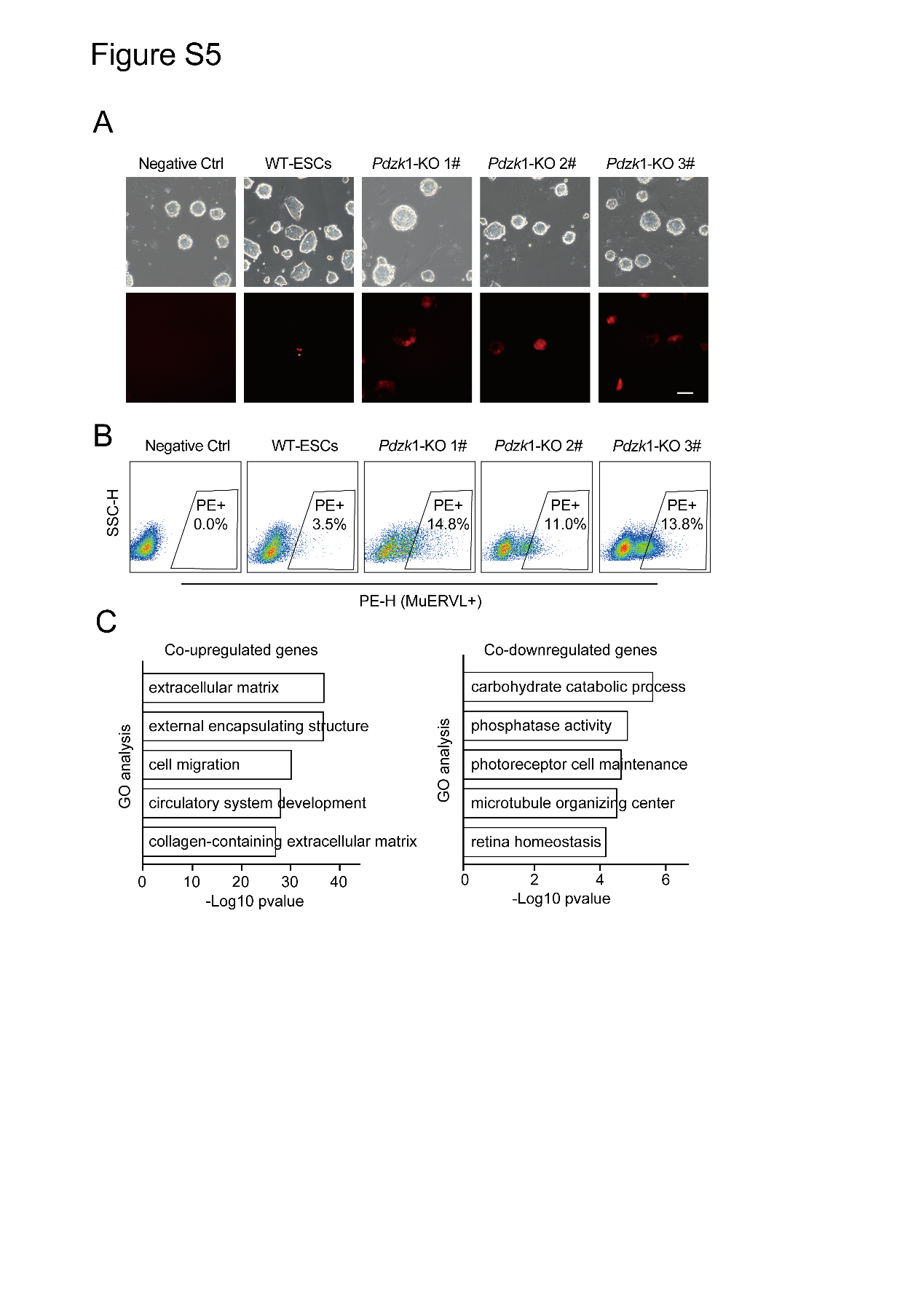


**Figure S5. The properties of** ***Pdzk1*-KO ESCs**

1. Images of *Pdzk1*-KO ESCs in BF and TRITC channels. All ESCs carried a 2C:tdTomato reporter (MuERVL+), with a non-labelled one as a negative control. Scale bar, 100 μm.
2. FACS analysis of RFP + (MuERVL+) cells in *Pdzk1*-KO ESCs and WT-ESCs. The percentage of RFP+ cells in *Pdzk1*-KO ESCs ranged from 11.0% to 14.8%, while that in WT-ESCs was only 3.5%.
3. GO analysis of co-upregulated and co-downregulated DEGs among *Pdzk1*-KO ESCs, *Rif1*-KO ESCs and *Dyrk1a*-KO ESCs, when vs. WT-ESCs.


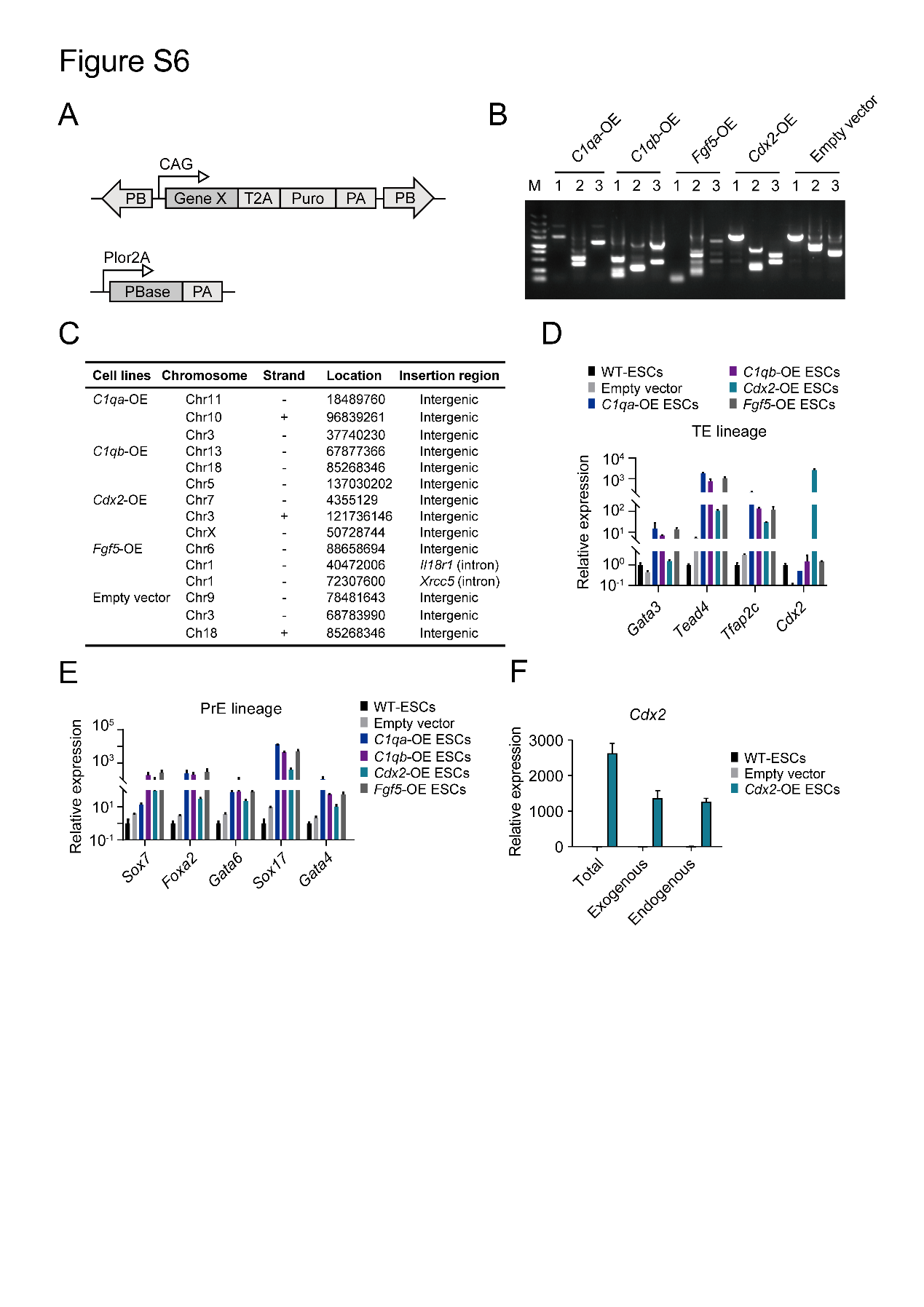


**Figure S6. Validation experiments for lineage inducers**

1. Schematic diagram of overexpression vector designing.
2. The inverse PCR results of PB insertions in *C1qa*-OE, *C1qb*-OE, *Fgf5*-OE and *Cdx2*-OE ESCs sub-clones.
3. The results of Sanger sequencing indicate that all the checked insertion sites are in inactive regions of the genome.
4. Expression levels of TE markers in *C1qa*-OE, *C1qb*-OE, *Fgf5*-OE and *Cdx2*-OE ESCs, compared with those in the Empty vector group and WT-ESCs.
5. Expression levels of PrE markers in *C1qa*-OE, *C1qb*-OE, *Fgf5*-OE and *Cdx2*-OE ESCs, compared with those in the Empty vector group and WT-ESCs.
6. Expression levels of total, exogenous and endogenous *Cdx2* in *Cdx2*-OE ESCs, compared with those in the Empty vector group and WT-ESCs.


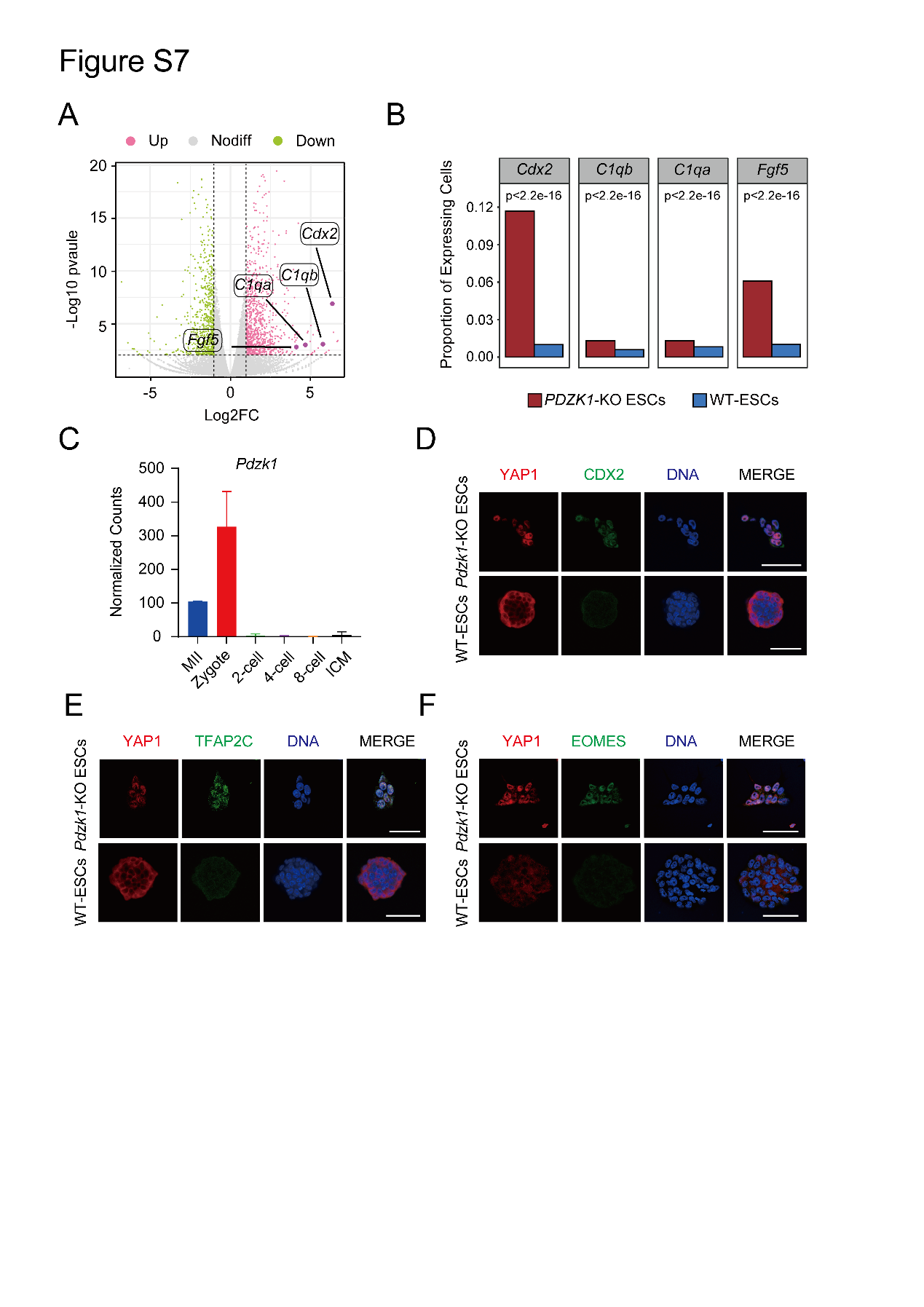


**Figure S7. Molecular characteristics of *Pdzk1*-KO ESCs**

1. Volcano plot of DEGs between *Pdzk1*-KO ESCs and WT-ESCs. *C1qa*, *C1qb*, *Fgf5* and *Cdx2* are highlighted.
2. The percentages of cells expressing *Cdx2*, *C1qb*, *C1qa* and *Fgf5* in *Pdzk1*-KO ESCs and WT-ESCs according to single-cell analysis.
3. The expression levels of *Pdzk1* in different stages of early embryonic development.
4. Immunofluorescence staining of YAP1 (red) and CDX2 (green) in *Pdzk1*-KO ESCs and WT-ESCs. DNA is stained with Hoechst 33342 (blue). Scale bar, 50 μm.
5. Immunofluorescence staining of YAP1 (red) and TFAP2C (green) in *Pdzk1*-KO ESCs and WT-ESCs. DNA is stained with Hoechst 33342 (blue). Scale bar, 50 μm.
6. Immunofluorescence staining of YAP1 (red) and EOMES (green) in *Pdzk1*-KO ESCs and WT-ESCs. DNA is stained with Hoechst 33342 (blue). Scale bar, 50 μm.

**Table S1. Primer Sequences**

| *Pdzk1*-KO | Sg1-1 | caccgcctcctcaatcacccggatc |
| --- | --- | --- |
|  | Sg1-2 | aaacgatccgggtgattgaggaggc |
|  | Sg2-1 | caccgaatgataagaaaccgggccc |
|  | Sg2-2 | aaacgggcccggtttcttatcattc |
|  | *Pdzk1*-Genotype-F | cagggattctcaagtatgctcctc |
|  | *Pdzk1*-Genotype-R | cagggattctcaagtatgctcctc |
| qPCR | *Gapdh*-F | aggtcggtgtgaacggatttg |
|  | *Gapdh*-R | tgtagaccatgtagttgaggtca |
|  | *C1qa*-F | aaaggcaatccaggcaatatca |
|  | *C1qa*-R | tggttctggtatggactctcc |
|  | *C1qb*-F | gaggtctggacacacctgtta |
|  | *C1qb*-R | ctcccctttaatccctggagt |
|  | *Fgf5*-F | gaagcgtctcactcccgaag |
|  | *Fgf5*-R | gaagaaaacgtcgcgctact |
|  | *Cdx2*-total-F | gtccctaggaagccaagtgaa |
|  | *Cdx2*-total-R | ttggctctgcggttctgaaa |
|  | *Cdx2*-exo-F | CTTGAGTCCTGTGACCTCCTT |
|  | *Cdx2*-exo-R | CGGTCATTGGGCCAGGATTC |
|  | *Gata3*-F | ctcggccattcgtacatggaa |
|  | *Gata3*-R | ggatacctctgcaccgtagc |
|  | *Tead4*-F | tccgccaaatctatgacaagttc |
|  | *Tead4*-R | cgatgttggtattgaggtctgc |
|  | *Tfap2c*-F | atccctcacctctcctctcc |
|  | *Tfap2c*-R | ccagatgcgagtaatggtcgg |
|  | *Sox7*-F | caaggatgagaggaaacgtc |
|  | *Sox7*-R | ctctgcctcatccacatagg |
|  | *Foxa2*-F | agccccaacaagatgctgac |
|  | *Foxa2*-R | tggttgaaggcgtaatggtg |
|  | *Gata6*-F | gccgggagcaccagtaca |
|  | *Gata6*-R | gtgacagttggcacaggacag |
|  | *Sox17*-F | gccaaagacgaacgcaagc |
|  | *Sox17*-R | tcatgcgcttcacctgcttg |
|  | *Gata4*-F | tctcactatgggcacagcag |
|  | *Gata4*-R | aggaccaggctgttccaaga |
| Overexpression | *C1qa*-OE-F | nnnnnnacgcgtatggagacctctcagggatg |
|  | *C1qa*-OE-R | nnnnnngtcgacggccgaggggaaaatgagga |
|  | *C1qb*-OE-F | nnnnnnacgcgtatgaagacacagtggggtga |
|  | *C1qb*-OE-R | nnnnnngtcgaccgcatccatgtcagggaaaa |
|  | *Fgf5*-OE-F | nnnnnnacgcgtatgagcctgtccttgctctt |
|  | *Fgf5*-OE-R | nnnnnngtcgactccgtaaatttggcttaaca |
|  | *Cdx2*-OE-F | nnnnnnacgcgtatgtacgtgagctaccttct |
|  | *Cdx2*-OE-R | nnnnnngtcgacctgggtgacagtggagttta |
| Inverse PCR | 1st 3’ PB-F | CCTCGATATACAGACCGATAAAACACATG |
|  | 1st 3’ PB-R | TCTTCTATAAAGTAACAAAACTTTTATGAGGGACAGC |
|  | 1st 5’ PB-F | GGTCATAGGGCCGGGATTC |
|  | 1st 5’ PB-R | GACTGAGATGTCCTAAATGCACAGC |
|  | 2nd 3’ PB-F | CGCATGATTATCTTTAACGTACGTCAC |
|  | 2nd 3’ PB-R | GGATACGGGGAAAAGGCCTC |
|  | 2nd 5’ PB-F | TCTCCTCCACGTCACCGC |
|  | 2nd 5’ PB-R | GAGCAATATTTCAAGAATGCATGCGTC |
